# A local human Vδ1 T cell population is associated with survival in nonsmall-cell lung cancer

**DOI:** 10.1038/s43018-022-00376-z

**Published:** 2022-05-30

**Authors:** Yin Wu, Dhruva Biswas, Ieva Usaite, Mihaela Angelova, Stefan Boeing, Takahiro Karasaki, Selvaraju Veeriah, Justyna Czyzewska-Khan, Cienne Morton, Magdalene Joseph, Sonya Hessey, James Reading, Andrew Georgiou, Maise Al-Bakir, TRACERx Consortium, Nicolai J. Birkbak, Gillian Price, Mohammed Khalil, Keith Kerr, Shirley Richardson, Heather Cheyne, Tracey Cruickshank, Gareth A. Wilson, Rachel Rosenthal, Hugo Aerts, Madeleine Hewish, Girija Anand, Sajid Khan, Kelvin Lau, Michael Sheaff, Peter Schmid, Louise Lim, John Conibear, Roland Schwarz, Tom L. Kaufmann, Matthew Huska, Jacqui Shaw, Joan Riley, Lindsay Primrose, Dean Fennell, Allan Hackshaw, Yenting Ngai, Abigail Sharp, Oliver Pressey, Sean Smith, Nicole Gower, Harjot Kaur Dhanda, Kitty Chan, Sonal Chakraborty, Kevin Litchfield, Krupa Thakkar, Jonathan Tugwood, Alexandra Clipson, Caroline Dive, Dominic Rothwell, Alastair Kerr, Elaine Kilgour, Fiona Morgan, Malgorzata Kornaszewska, Richard Attanoos, Helen Davies, Katie Baker, Mathew Carter, Colin R. Lindsay, Fabio Gomes, Fiona Blackhall, Lynsey Priest, Matthew G. Krebs, Anshuman Chaturvedi, Pedro Oliveira, Zoltan Szallasi, Gary Royle, Catarina Veiga, Marcin Skrzypski, Roberto Salgado, Miklos Diossy, Alan Kirk, Mo Asif, John Butler, Rocco Bilancia, Nikos Kostoulas, Mathew Thomas, Mairead MacKenzie, Maggie Wilcox, Apostolos Nakas, Sridhar Rathinam, Rebecca Boyles, Mohamad Tufail, Amrita Bajaj, Keng Ang, Mohammed Fiyaz Chowdhry, Michael Shackcloth, Julius Asante-Siaw, Angela Leek, Nicola Totten, Jack Davies Hodgkinson, Peter Van Loo, William Monteiro, Hilary Marshal, Kevin G. Blyth, Craig Dick, Charles Fekete, Eric Lim, Paulo De Sousa, Simon Jordan, Alexandra Rice, Hilgardt Raubenheimer, Harshil Bhayani, Morag Hamilton, Lyn Ambrose, Anand Devaraj, Hemangi Chavan, Sofina Begum, Silviu I. Buderi, Daniel Kaniu, Mpho Malima, Sarah Booth, Andrew G. Nicholson, Nadia Fernandes, Pratibha Shah, Chiara Proli, John Gosney, Sarah Danson, Jonathan Bury, John Edwards, Jennifer Hill, Sue Matthews, Yota Kitsanta, Jagan Rao, Sara Tenconi, Laura Socci, Kim Suvarna, Faith Kibutu, Patricia Fisher, Robin Young, Joann Barker, Fiona Taylor, Kirsty Lloyd, Jason Lester, Mickael Escudero, Aengus Stewart, Andrew Rowan, Jacki Goldman, Richard Kevin Stone, Tamara Denner, Emma Nye, Maria Greco, Jerome Nicod, Clare Puttick, Katey Enfield, Emma Colliver, Alastair Magness, Chris Bailey, Krijn Dijkstra, Vittorio Barbè, Roberto Vendramin, Judit Kisistok, Mateo Sokac, Jonas Demeulemeester, Elizabeth Larose Cadieux, Carla Castignani, Hongchang Fu, Kristiana Grigoriadis, Claudia Lee, Foteini Athanasopoulou, Crispin Hiley, Lily Robinson, Tracey Horey, Peter Russell, Dionysis Papadatos-Pastos, Sara Lock, Kayleigh Gilbert, Kayalvizhi Selvaraju, Paul Ashford, Oriol Pich, Thomas B. K. Watkins, Sophia Ward, Emilia Lim, Alexander M. Frankell, Christopher Abbosh, Robert E. Hynds, Mariana Werner Sunderland, Karl Peggs, Teresa Marafioti, John A. Hartley, Helen Lowe, Leah Ensell, Victoria Spanswick, Angeliki Karamani, David Moore, Stephan Beck, Olga Chervova, Miljana Tanic, Ariana Huebner, Michelle Dietzen, James R. M. Black, Carlos Martinez Ruiz, Robert Bentham, Cristina Naceur-Lombardelli, Haoran Zhai, Nnennaya Kanu, Francisco Gimeno-Valiente, Supreet Kaur Bola, Ignacio Garcia Matos, Mansi Shah, Felipe Galvez Cancino, Despoina Karagianni, Maryam Razaq, Mita Akther, Diana Johnson, Joanne Laycock, Elena Hoxha, Benny Chain, David R. Pearce, Kezhong Chen, Javier Herrero, Fleur Monk, Simone Zaccaria, Neil Magno, Paulina Prymas, Antonia Toncheva, Monica Sivakumar, Olivia Lucas, Mark S. Hill, Othman Al-Sawaf, Seng Kuong Ung, Sam Gamble, Sophia Wong, David Lawrence, Martin Hayward, Nikolaos Panagiotopoulos, Robert George, Davide Patrini, Mary Falzon, Elaine Borg, Reena Khiroya, Asia Ahmed, Magali Taylor, Junaid Choudhary, Sam M. Janes, Martin Forster, Tanya Ahmad, Siow Ming Lee, Neal Navani, Marco Scarci, Pat Gorman, Elisa Bertoja, Robert C. M. Stephens, Emilie Martinoni Hoogenboom, James W. Holding, Steve Bandula, Ricky Thakrar, James Wilson, Mansi Shah, Marcos, Vasquez Duran, Maria Litovchenko, Sharon Vanloo, Piotr Pawlik, Kerstin Thol, Babu Naidu, Gerald Langman, Hollie Bancroft, Salma Kadiri, Gary Middleton, Madava Djearaman, Aya Osman, Helen Shackleford, Akshay Patel, Christian Ottensmeier, Serena Chee, Aiman Alzetani, Judith Cave, Lydia Scarlett, Jennifer Richards, Papawadee Ingram, Emily Shaw, John Le Quesne, Alan Dawson, Domenic Marrone, Sean Dulloo, Claire Wilson, Yvonne Summers, Raffaele Califano, Rajesh Shah, Piotr Krysiak, Kendadai Rammohan, Eustace Fontaine, Richard Booton, Matthew Evison, Stuart Moss, Juliette Novasio, Leena Joseph, Paul Bishop, Helen Doran, Felice Granato, Vijay Joshi, Elaine Smith, Angeles Montero, Phil Crosbie, Nicholas McGranahan, Mariam Jamal-Hanjani, Allan Hackshaw, Sergio A. Quezada, Adrian C. Hayday, Charles Swanton

**Affiliations:** 1 Cancer Research UK Lung Cancer Centre of Excellence, University College London Cancer Institute, London, UK; 2 Cancer Evolution and Genome Instability Laboratory, The Francis Crick Institute, London, UK; 3 Peter Gorer Department of Immunobiology, School of Immunology & Microbial Sciences, King’s College London, London, UK; 4 Immunosurveillance Laboratory, The Francis Crick Institute, London, UK; 5 Bill Lyons Informatics Centre, University College London Cancer Institute, London, UK; 6 Bioinformatics & Biostatistics and Software Development & Machine Learning Team, The Francis Crick Institute, London, UK; 7 Cancer Metastasis Lab, University College London Cancer Institute, London, UK; 8 Cancer Immunology Unit, Research Department of Haematology, University College London Cancer Institute, London, UK; 9 Cancer Genome Evolution Research Group, University College London Cancer Institute, London, UK; 10 Cancer Research UK & University College London Cancer Trials Centre, University College London, London, UK; 11 Aarhus University, Aarhus, Denmark; 12 Aberdeen Royal Infirmary, Aberdeen, UK; 13 Achilles Therapeutics UK Limited, London, UK; 14 Artificial Intelligence in Medicine Program, Harvard Medical School, Boston, MA USA; 15 Radiology and Nuclear Medicine, CARIM & GROW, Maastricht University, Maastricht, The Netherlands; 16 Ashford and St. Peter’s Hospitals NHS Foundation Trust, Chertsey, UK; 17 Barnet & Chase Farm Hospitals, London, UK; 18 Barts Health NHS Trust, London, UK; 19 Berlin Institute for Medical Systems Biology, Max Delbrück Center for Molecular Medicine in the Helmholtz Association, Berlin, Germany; 20 Cancer Research Centre, University of Leicester, Leicester, UK; 21 Leicester University Hospitals, Leicester, UK; 22 Cancer Research UK & UCL Cancer Trials Centre, London, UK; 23 Cancer Research UK Manchester Institute, University of Manchester, Manchester, UK; 24 Cancer Research UK Lung Cancer Centre of Excellence, University of Manchester, Manchester, UK; 25 Cardiff & Vale University Health Board, Cardiff, UK; 26 Christie NHS Foundation Trust, Manchester, UK; 27 Danish Cancer Society Research Centre, Copenhagen, Denmark; 28 Department of Medical Physics and Bioengineering, University College London Cancer Institute, London, UK; 29 Department of Oncology and Radiotherapy, Medical University of Gdańsk, Gdańsk, Poland; 30 Department of Pathology, GZA-ZNA Antwerp, Antwerp, Belgium; 31 Department of Physics of Complex Systems, ELTE Eötvös Loránd University, Budapest, Hungary; 32 Golden Jubilee National Hospital, Clydebank, UK; 33 Independent Cancer Patients Voice, London, UK; 34 Liverpool Heart and Chest Hospital NHS Foundation Trust, Liverpool, UK; 35 Manchester Cancer Research Centre Biobank, Manchester, UK; 36 MD Anderson Cancer Center, Houston, TX USA; 37 National Institute for Health Research, Leicester Respiratory Biomedical Research Unit, Leicester, UK; 38 NHS Greater Glasgow and Clyde, Glasgow, UK; 39 Royal Brompton and Harefield NHS Foundation Trust, London, UK; 40 Royal Liverpool University Hospital, Liverpool, UK; 41 Sheffield Teaching Hospitals NHS Foundation Trust, Sheffield, UK; 42 Swansea Bay University Health Board, Swansea, UK; 43 The Francis Crick Institute, London, UK; 44 University College London Medical School, London, UK; 45 The Princess Alexandra Hospital NHS Trust, Harlow, UK; 46 The Whittington Hospital NHS Trust, London, UK; 47 University College London Cancer Institute, London, UK; 48 University College London Hospitals, London, UK; 49 University Hospital Birmingham NHS Foundation Trust, Birmingham, UK; 50 University Hospital Southampton NHS Foundation Trust, Southampton, UK; 51 Institute of Cancer Sciences, University of Glasgow, Glasgow, UK; 52 University of Leicester, Leicester, UK; 53 Wythenshawe Hospital, Manchester University NHS Foundation Trust, Manchester, UK; 54 Division of Infection, Immunity and Respiratory Medicine, University of Manchester, Manchester, UK

**Keywords:** Gammadelta T cells, Cancer, Non-small-cell lung cancer, Tumour immunology

## Abstract

Murine tissues harbor signature γδ T cell compartments with profound yet differential impacts on carcinogenesis. Conversely, human tissue-resident γδ cells are less well defined. In the present study, we show that human lung tissues harbor a resident Vδ1 γδ T cell population. Moreover, we demonstrate that Vδ1 T cells with resident memory and effector memory phenotypes were enriched in lung tumors compared with nontumor lung tissues. Intratumoral Vδ1 T cells possessed stem-like features and were skewed toward cytolysis and helper T cell type 1 function, akin to intratumoral natural killer and CD8^+^ T cells considered beneficial to the patient. Indeed, ongoing remission post-surgery was significantly associated with the numbers of CD45RA^−^CD27^−^ effector memory Vδ1 T cells in tumors and, most strikingly, with the numbers of CD103^+^ tissue-resident Vδ1 T cells in nonmalignant lung tissues. Our findings offer basic insights into human body surface immunology that collectively support integrating Vδ1 T cell biology into immunotherapeutic strategies for nonsmall cell lung cancer.

## Abstract (web summary)

Wu et al. utilize multiparametric analysis of early-stage human NSCLC to characterize a population of Vδ1 T cells displaying a resident memory and effector memory phenotype, which were associated with ongoing remission.

## Main

Immune checkpoint inhibitors (CPIs) have revolutionized the treatment of cancer by providing durable remissions, albeit for a minority of patients. CPIs work, at least in part, by de-repressing tumor (neo)antigen-specific αβ T cells. Thus, many efforts to improve efficacy have focused on this axis^1,2^. Indeed, some utility of tumor mutational burden (TMB)^3,4^, presence and quality of CD8^+^ T cells^5,6^ and major histocompatibility complex (MHC) class I loss of heterozygosity^7^ in predicting responses to CPIs provide collective evidence of the contributions of antigen-specific αβ T cells. Nevertheless, a high TMB does not guarantee responses to CPIs and a lack of MHC-I and/or low TMB does not preclude good responses^8,9^.

Alongside αβ T cells, we and others have previously demonstrated that γδ T cells are also found within tumors^10–14^. These cells are evolutionarily conserved, implying a vital and nonredundant role and, although they are often less abundant than αβ T cells, this disparity may be compensated by their polyclonal response potentials compared with the highly clonotypic responses of αβ T cells^15^. Indeed, it is well established that γδ T cells protect against cancer in mice, both independently and synergistically with αβ T cells^16,17^, highlighting the need to better assess their importance in human cancers^18,19^. Although such studies have been limited by the availability of technologies to rigorously identify, isolate and examine γδ T cells, a recent in silico study of >5,000 cancer patients demonstrated that, of all 22 immune cell types studied, the intratumoral γδ T cell signature was the trait most significantly associated with remission^10^.

Similar to αβ T cells, γδ T cells are also composed of distinct subsets occupying different functional niches. It is clear in the murine setting that body surface tissues in which carcinomas arise harbor signature tissue-specific γδ T cell subsets^20^. We recently demonstrated that human breast epithelium is enriched in the Vδ1 subset of γδ T cells and that these cells possess potent anti-tumor functions, including the capacity to kill transformed cells in vitro and produce tumor-antagonistic cytokines, for example, interferon-γ (IFN-γ)^11^. Moreover, activation of these Vδ1 T cells did not require cognate peptide–MHC but instead the cells responded to conserved signals of tissue stress, via innate receptors, entirely distinct from co-located αβ T cells^11^. In other words, Vδ1 T cells were an independent, but potentially synergistic, population of anti-tumor lymphocytes in the tumor microenvironment (TME). Furthermore, in patients with aggressive triple-negative breast cancers (TNBCs), the intratumoral presence of Vδ1 T cells was more significantly associated with survival than co-located αβ T cells. A subsequent study employing CIBERSORT demonstrated that intratumoral γδ T cells also predicted survival in a larger cohort of 169 patients with TNBCs in the METABRIC dataset^21^. Recent studies have described similar populations of resident Vδ1 T cells in colorectal cancers, where their presence was associated with lower-stage disease^12^, and in hepatocellular carcinomas, where the presence of total γδ T cells was associated with survival^14^. Thus, we hypothesize that human body surface tissues, similar to their murine counterparts, harbor signature tissue-resident γδ T cells contributing to cancer immunosurveillance.

To test this hypothesis in human lung, we have leveraged samples and clinical data collected from the TRACERx (Tracking non-small-cell lung Cancer Evolution through therapy (Rx)) Study^22^. Nonsmall cell lung cancers (NSCLCs) are cancers of unmet clinical need. Although outcomes are better for patients with early-stage disease, overall outcome is poor with a 5-year survival rate of <20%^23^. These cancers are clearly susceptible to immunosurveillance, as witnessed by instances of successful CPI therapy and evidence of immune editing in treatment-naive primary lung cancers^24^. However, despite harboring high TMBs^25^, only a minority of patients with NSCLCs respond to CPI therapy. By employing flow cytometry, quantitative T cell receptor-sequencing (TCR-seq) and RNA-sequencing (RNA-seq), we now find that both nontumor (NT) human lung tissues and NSCLCs harbor resident populations of γδ T cells, particularly enriched in the Vδ1 subtype. Moreover, these Vδ1 T cells possess a T-cytolytic type 1 (Tc1) phenotype that is well established as beneficial to patients with cancer^26–28^. Finally, the presence of Vδ1 T cells in both NT tissue and lung tumors was significantly associated with remission. The former association is of particular interest because cell populations in NT tissue, as opposed to tumors, may be retained in situ postoperatively.

## Results

### Vδ1 T cells are present in lung epithelium and enriched in NSCLCs

**Fig. 1:**
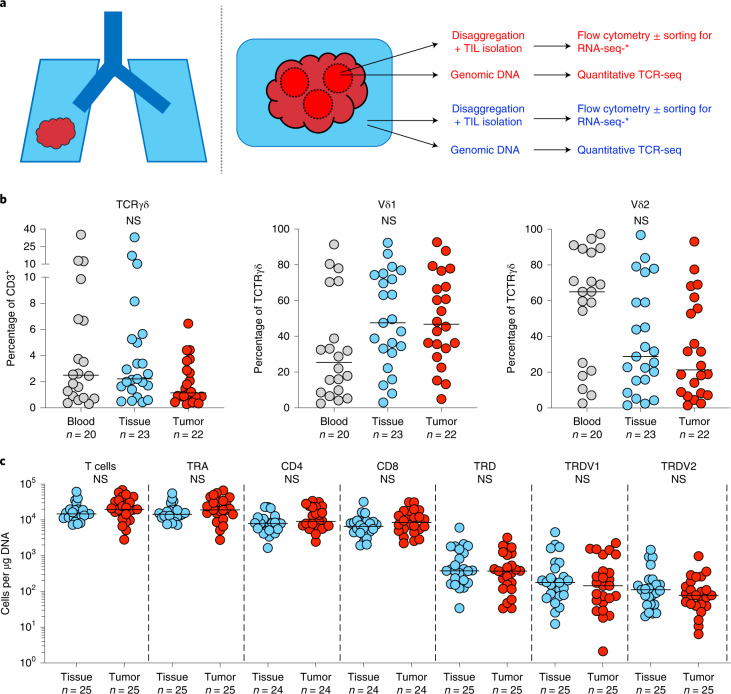
Experimental design and γδ T cell composition in lung tissues and NSCLCs. **a**, Overview of study design. Paired tumor regions (red) and NT lung tissues (blue) collected under the TRACERx Study were enzymatically digested to extract tissue/TILs. TILs were cryopreserved and thawed at a later date for flow cytometry ± RNA-seq. In parallel, gDNA was extracted from undigested matched tumor regions and NT lung tissues and sent for subsequent quantitative TCR-seq. In addition, PBMCs were isolated from contemporaneous blood draws and cryopreserved before subsequent thaw for flow cytometry. **b**, Percentage of CD3^+^ T cells staining for TCRγδ (left) and percentage of TCRγδ T cells staining for Vδ1 (middle) and Vδ2 (right) in PBMCs (blood), NT lung tissues (tissue) and tumors (tumor). Not all patients had paired samples. The bar represents the median. The Kruskal–Wallis test with post-hoc Dunn’s test corrected for multiple testing was used. **c**, Absolute counts of total T cells, αβ T cells (TRA), γδ T cells (TRD) and Vδ1 (*TRDV1*) and Vδ2 (*TRDV2*) T cells per microgram of DNA determined by TCR-seq. Absolute counts of CD4^+^ αβ T cells (CD4) and CD8^+^ αβ T cells (CD8) were determined by mapping the proportion of CD3^+^/TCRγδ^−^ T cells staining for CD4 or CD8 in flow cytometry analysis of paired TILs. No significant differences were observed within demarcated T cell subsets between NT tissues and tumors. Samples with <1 cell μg^−1^ of DNA were not plotted for the purposes of visualization. The bar represents the median. A two-tailed Mann–Whitney *U*-test was used within demarcated T cell subsets. Significant *P* values are shown. NS, not significant. The *n* numbers and datapoints represent independent patients. Source data

To characterize the T cell landscape in human lung epithelium and NSCLCs, we examined tissue and tumor-infiltrating lymphocytes (TILs) isolated from NT lung tissues and paired tumors of patients with surgically resected NSCLCs collected from the TRACERx Study (Supplementary Table 1). NT tissues were taken as far as possible from tumors at primary surgery and hematoxylin and eosin (H&E)-stained sections examined afterwards by an accredited histopathologist to ensure that samples were tumor free. TILs were isolated by enzymatic digestion and cryopreserved before thawing for use in downstream assays without further manipulation or expansion (see Methods). TILs were immunophenotyped by flow cytometry and absolute T cell counts established using quantitative TCR-seq of region-matched genomic (g)DNA extracted from bulk tissues and tumors (Fig. 1a). Samples were chosen based on the availability of banked TILs and region-matched bulk DNA from patients with at least one follow-up visit after surgical resection (see Methods). Where available, paired contemporaneous peripheral blood mononuclear cells (PBMCs) were also immunophenotyped by flow cytometry to contextualize findings with the well-characterized blood γδ T cell compartment. No other selection criteria were applied.

**Extended Data Fig. 1:**
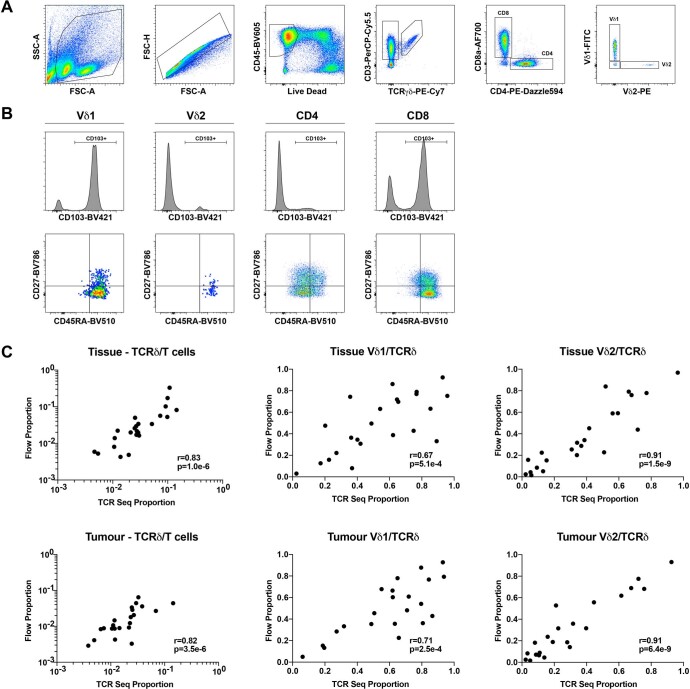
Lung γδ T cells assayed by flow cytometry and TCR sequencing. **A)** Representative flow cytometry gating strategy for T cell subsets. From left to right: Forward vs. side scatter gate -> singlet gate -> live CD45 + gate -> CD3 vs. TCRγδ -> CD4 vs. CD8 (of CD3 + /TCRγδ- population) and Vδ1 vs. Vδ2 (of CD3 + / TCRγδ + population) to give CD4 and CD8 αβ T cells and Vδ1 and Vδ2 γδ T cells. **B)** Representative gating strategy for CD103 and CD45RA vs. CD27 for Vδ1 and Vδ2 γδ T cells, and CD4 and CD8 αβ T cells. **C)** Proportion of T cell subsets determined by flow cytometry versus proportion determined by TCRseq in NT tissues (n = 23 patients) and tumours (n = 22 patients). Two-tailed Spearman correlation. Significant p values shown. All datapoints represent independent patients. Source data

**Extended Data Fig. 2:**
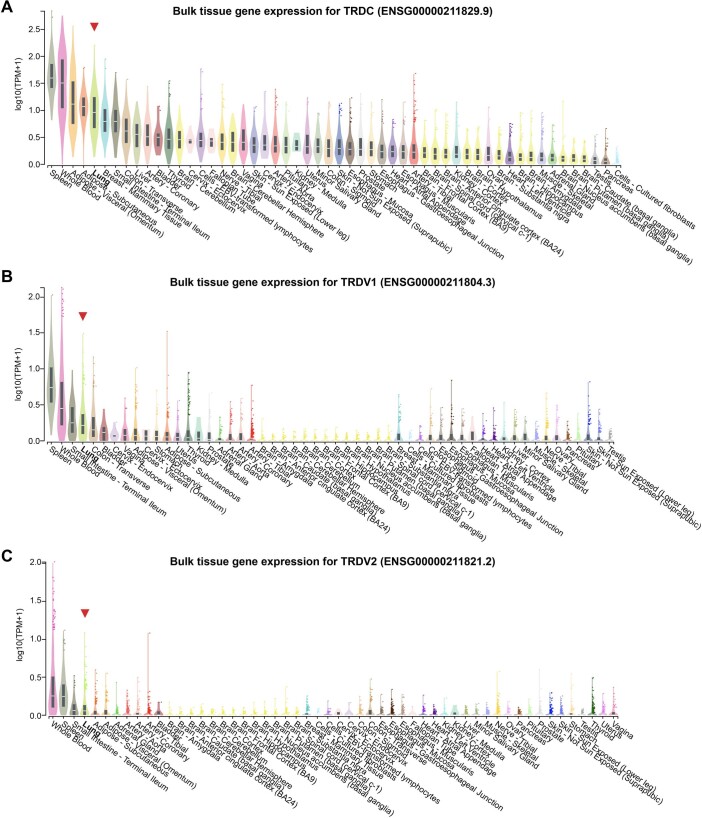
TCRδ is expressed in non-diseased lung tissues. **A)** Expression of *TRDC* across non-diseased tissue types. **B)** Expression of *TRDV1* across non-diseased tissue types. **C)** Expression of *TRDV2* across non-diseased tissue types. Gene expression data and figures derived from the GTEx Project and Portal (see methods). Lung expression (red arrow) based on 578 donors. Boxplots represent median and interquartile range with outliers (above or below 1.5x IQR) displayed as points. Tissues ordered from highest to lowest median expression of each gene. Datapoints represent independent donors.

Similar to their presence in peripheral blood (median, interquartile range (IQR): 2.49%, 0.80–6.76%), γδ T cells detected by flow cytometry comprised a small fraction of total T cells in both NT lung tissues (2.22%, 1.39–5.26%) and tumors (1.15%, 0.85–3.05%), with considerable interindividual variation (Fig. 1b). In contrast to peripheral blood, where most γδ T cells expressed Vδ2, most γδ T cells found in lung tissues and tumors expressed Vδ1 (47.6%, 30.8–74.4% and 46.8%, 32.1–70.0% respectively), consistent with a well-established enrichment of Vδ1 T cells in other tissues^29^ (Fig. 1b and Extended Data Fig. 1a,b). Complementary to flow cytometry, we employed quantitative TCR-seq of gDNA at the TCRα/δ (TRA/TRD) locus from matched NT tissues and tumor regions as an independent assay of αβ and γδ T cells, and to assess their absolute numbers per unit of tissue (Fig. 1c). Indeed, there was a strong and significant correlation between the proportion of major Vδ subsets (Vδ1 and Vδ2) detected by flow cytometry and TCR-seq (Extended Data Fig. 1c). We then mapped flow phenotyping proportions (that is, proportion of CD3^+^ T cells positive for CD4^+^/CD8^+^) on to absolute TCR-seq counts of T cells to derive more granular T cell subset numbers (Fig. 1c). Consistent with flow cytometry, TCR-seq also revealed that γδ T cells form a minority subset of total T cells in NT lung tissues (2.70%, 1.30–6.23%) and tumors (1.68%, 0.97–2.60%), and that the Vδ1 subset comprises most of these cells in both NT lung tissues (54.2%, 35.9–76.7%) and tumors (62.0%, 29.5–79.6%) (Fig. 1c). Although NT lung tissues were macroscopically and microscopically tumor free, it was not practical to obtain normal lung tissue from healthy donors to exclude potential effects of tumors on the immune microenvironment of NT lung tissues. To address this issue, we utilized data generated by the Genotype-Tissue Expression (GTEx) project^30^, a comprehensive public resource of tissue-specific gene expression from 54 nondiseased tissue sites across almost 1,000 individuals. We found that *TRDC*, a gene expressed by all γδ T cells, was highly expressed in lung tissues compared with other tissue sites (Extended Data Fig. 2a). Furthermore, expression of *TRDV1* (Vδ1 T cells) was higher than *TRDV2* (Vδ2 T cells) within lung tissue (Extended Data Fig. 2b,c), consistent with our own data. Thus, we conclude that γδ T cells, particularly the Vδ1 subset, are present in nonmalignant lung tissue at a steady state.

**Fig. 2:**
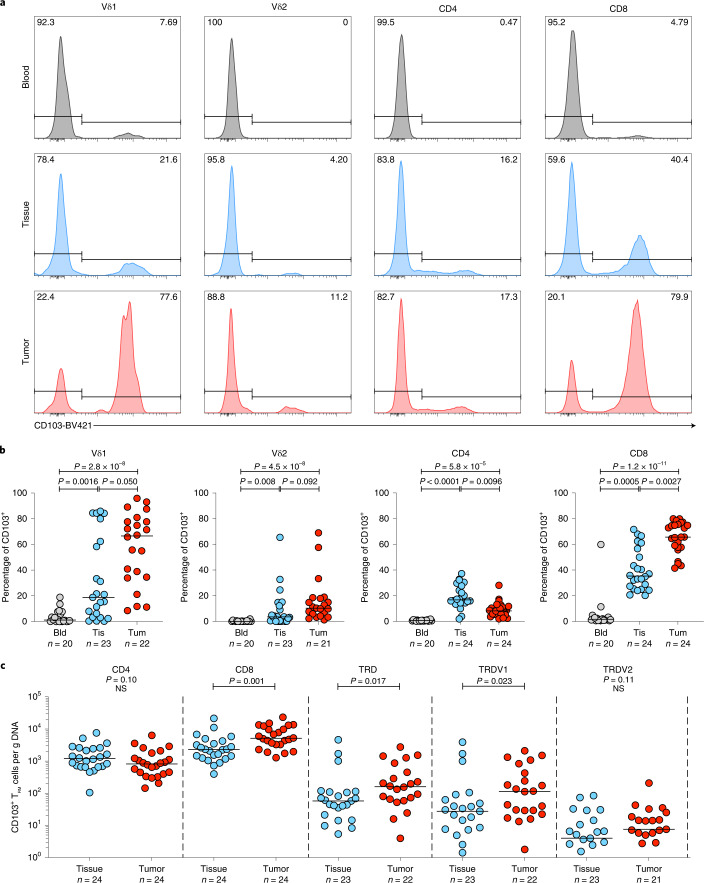
NT lung tissue harbors tissue-resident Vδ1T cells that are enriched in NSCLCs. **a**, Representative plots of CD103 expression (%) by flow cytometry on Vδ1 (representing *n* = 20, *n* = 23 and *n* = 22 patients for blood, NT tissue and tumor, respectively) and Vδ2 (representing *n* = 20, *n* = 23 and *n* = 21 patients for blood, NT tissue and tumor, respectively), CD4^+^ (representing *n* = 20, *n* = 24 and *n* = 24 patients for blood, NT tissue and tumor, respectively) and CD8^+^ T cells (representing *n* = 20, *n* = 24 and *n* = 24 patients for blood, NT tissue and tumor, respectively) were isolated from blood, NT tissue and tumor of one patient. **b**, Summary flow cytometry data of CD103 expression in T cell subsets isolated from blood (Bld), NT tissues (Tis) and tumors (Tum). Not all patients had paired samples. The bar represents the median. A Kruskal–Wallis test with a post-hoc Dunn’s test corrected for multiple testing was used. **c**, Absolute counts of CD103^+^ T_RM_ CD4^+^, CD8^+^, Vδ1 and Vδ2 T cells per microgram of DNA from NT tissues and tumors. Samples with <1 cell μg^−1^ of DNA were not plotted for the purposes of visualization. Not all patients had paired samples. The bar represents the median. A two-tailed Mann–Whitney *U*-test was used within demarcated T cell subsets. Significant *P* values are shown. NS, not significant. The *n* numbers and datapoints represent independent patients. Source data

In many tissues, CD103 has been adopted as a marker of tissue-resident memory (T_RM_) status, absent on most peripheral blood T cells^31^. Consistent with previous studies, NT lung tissues harbored a CD103^+^CD8^+^TCRαβ T_RM_ population (median, IQR: 35.3%, 25.9–55.0%) (Fig. 2a,b)^32,33^. Similar to the CD8^+^ T cell compartment, we found that many, albeit not all, Vδ1 T cells in NT lung tissues displayed a CD103^+^ T_RM_ phenotype, particularly in some patients (18.8%, 3.35–62.4%) (Fig. 2a,b). In contrast, most CD4^+^ and Vδ2 T cells in NT lung tissues were CD103^−^ as were their counterparts in peripheral blood (Fig. 2a,b). Thus, the resident CD103^+^ T cell compartment in NT lung tissues mostly comprised CD8^+^ and Vδ1 T cells.

Compared with NT lung tissues, tumors harbored a greater proportion of CD8^+^ (median, IQR: 65.6%, 57.7–75.3%), Vδ1 (66.6%, 34.4–78.5%) and Vδ2 T cells (10.3%, 5.03–18.2%) expressing CD103 whereas the proportion of CD103 expressing CD4^+^ T cells was reduced (Fig. 2b). By mapping flow phenotyping proportions on to TCR-seq counts, we observed significantly greater absolute numbers of CD8^+^ and Vδ1 T cells with the CD103 phenotype in tumors compared with NT lung tissues; conversely, this was not so for CD4^+^ and Vδ2 T cells (Fig. 2c).

### Intratumoral Vδ1 T cells possess a Tc1 functional phenotype in situ

**Fig. 3:**
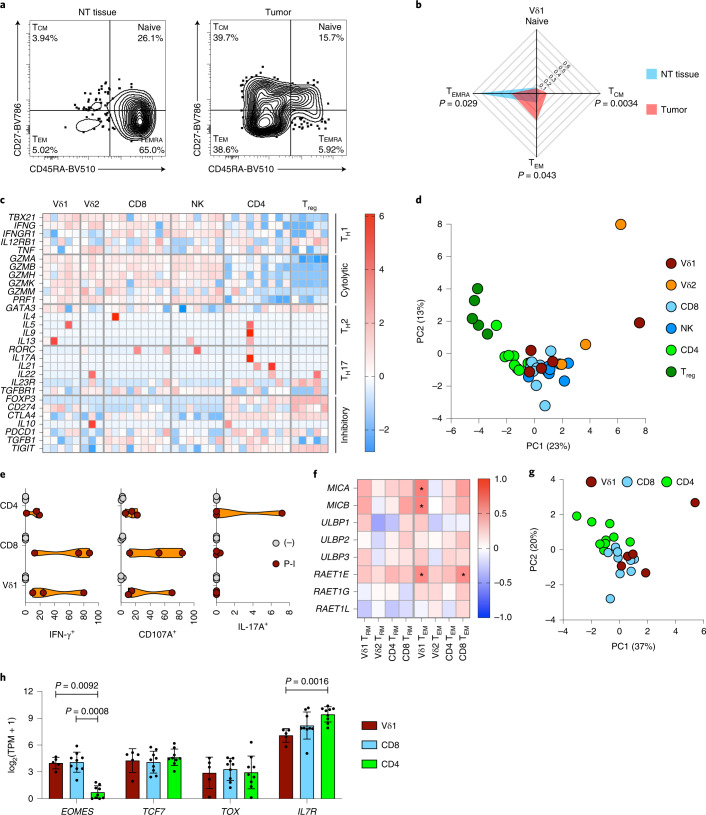
Intratumoral Vδ1 T cells have a memory phenotype, are Tc1 skewed and demonstrate features of tissue residency and stemness. **a**, Representative flow cytometry plots of effector memory status (defined by CD27 and CD45RA expression) of Vδ1 T cells isolated from the NT tissue and tumor of one patient (representing *n* = 23 and *n* = 22 patients for NT tissue and tumor, respectively). **b**, Summary radar plot of effector memory status of Vδ1 T cells isolated from NT tissues (*n* = 23) and tumors (*n* = 22). The median proportion is plotted. A two-tailed Mann–Whitney *U*-test was used between NT tissue and tumor within Vδ1 memory subsets. Significant *P* values are shown. **c**, Expression of T cell master transcription factors and signature effector molecules of lymphocytes sorted directly from tumors grouped into T_H_ cell, cytolytic and inhibitory modules. Each column represents the denoted cell type from an individual patient. Not all cell types were sorted from matched patients. The color scale denotes the *z*-score of log_2_(TPM + 1) of each gene. **d**, PCA of expression (normalized counts) of genes included in **c** colored by cell type (*n* = 9, *n* = 9, *n* = 7, *n* = 5, *n* = 3 and *n* = 5 patients for CD4^+^, CD8^+^, NK, T_reg_, Vδ2 and Vδ1 cells, respectively). **e**, Violin plots showing intracellular cytokine staining for IFN-γ and IL-17A and cell surface staining for CD107A in Vδ1, CD8^+^ and CD4^+^ T cells after in vitro stimulation of bulk TILs (*n* = 3 patients) with PMA and ionomycin (P-I). **f**, Summary data of correlation between region-matched gene expression of NKG2D ligands and absolute numbers of Vδ1 (*n* = 14 patients), Vδ2 (*n* = 14 patients), CD4^+^ (*n* = 15 patients) and CD8^+^ (*n* = 15 patients) T_RM_ and T_EM_ cells in tumors. The color scale denotes a two-tailed Spearman’s *r*. * denotes significant correlations as follows: *MICA*:Vδ1 T_EM_ *P* = 0.027, *MICB*:Vδ1 T_EM_ *P* = 0.031, *RAET1E*:Vδ1 T_EM_ *P* = 0.048 and *RAET1E*:CD8 T_EM_ *P* = 0.045. **g**, PCA of expression (normalized counts) of core T_RM_ gene signature (*CCR7, CD69, CXCR6, ITGAE, ITGA1, S1PR1* and *SELL*) in Vδ1 (*n* = 5 patients), CD4^+^ (*n* = 9 patients) and CD8^+^ T cells (*n* = 9 patients) sorted from tumors. **h**, Expression of genes that define ‘stem-like’ CD8^+^ T cells in Vδ1 (*n* = 5 patients), CD4^+^ (*n* = 9 patients) and CD8^+^ (*n* = 9 patients) T cells sorted from tumors. The mean ± s.d. is plotted. A Kruskal–Wallis test with post-hoc Dunn’s test correction for multiple testing was used. All datapoints represent independent patients. Source data

Intratumoral CD8^+^ T_RM_ cells have been associated with survival in several cancers^34,35^ including NSCLCs^36^. However, the potential contributions of human Vδ1 T cells are less well described and more contentious^37^. Similar to CD8^+^ αβ T cells, γδ T cells have been operationally classified into effector T cell/memory T cell subsets associated with defined effector functions based on CD45RA and CD27 expression^38^. When we compared Vδ1 T cells in tumors versus NT tissues, we observed an overt shift toward a CD45RA^−^CD27^−^ effector memory T cell (T_EM_) phenotype by flow cytometry (Fig. 3a,b). Similar to their CD8^+^ counterparts, Vδ1 T_EM_ cells have been associated with helper type 1 T cell (T_H_1 cell) cytokine production (IFN-γ) and cytolysis^38^, but whether intratumoral Vδ1 T cells possess this patient-beneficial Tc1 phenotype^27,28^ in situ has remained unclear.

**Extended Data Fig. 3:**
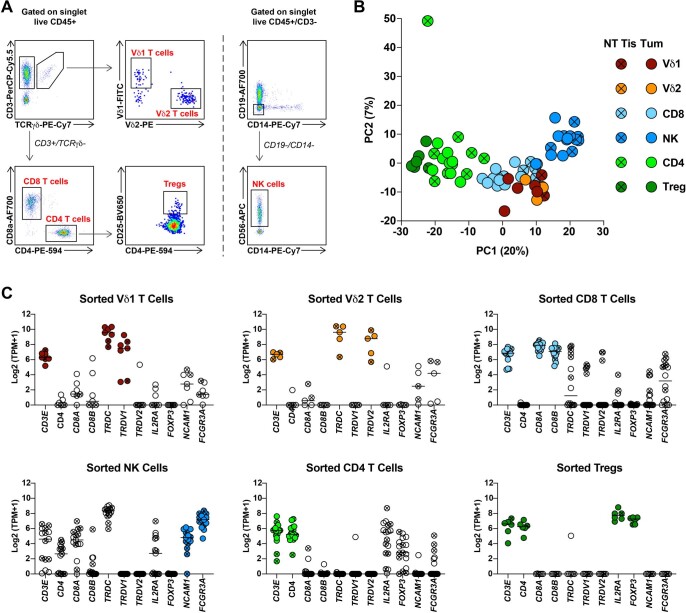
Lung TIL lineages by RNA sequencing. **A)** Gating strategy for sorting CD4, CD8, Vδ1 and Vδ2 T cells and Tregs and NK cells (red/bold) for bulk cell-type RNA sequencing. **B)** PCA of top 500 variable genes coloured by cell type from tumours (n = 9, n = 9, n = 7, n = 5, n = 3 and n = 5 patients for CD4^+^, CD8^+^, NK, Treg, Vδ2 and Vδ1 cells respectively) and NT tissue (n = 8, n = 7, n = 8, n = 1, n = 2 and n = 2 patients for CD4^+^, CD8^+^, NK, Treg, Vδ2 and Vδ1 cells respectively). **C)** Expression of canonical lineage markers from sorted cell types. Lineage markers for each sorted cell type coloured in. Cells sorted from NT tissues denoted by crossed-circles (n = 8, n = 7, n = 8, n = 1, n = 2 and n = 2 patients for CD4^+^, CD8^+^, NK, Treg, Vδ2 and Vδ1 cells respectively) and cells sorted from lung tumours denoted by solid circles (n = 9, n = 9, n = 7, n = 5, n = 3 and n = 5 patients for CD4^+^, CD8^+^, NK, Treg, Vδ2 and Vδ1 cells respectively). All datapoints represent independent patients. Source data

To address this, we sorted bulk Vδ1 T cells from disaggregated NT tissues and lung tumors (*n* = 2 and *n* = 5 patients, respectively) from which there was sufficient material. For comparison, we also sorted, from NT lung tissues and tumors, bulk Vδ2 (*n* = 3 and *n* = 3 patients), CD4^+^ (*n* = 8 and *n* = 9 patients), CD8^+^ (*n* = 7 and *n* = 9), and regulatory T cells (T_reg_ cells) (*n* = 1 and *n* = 5 patients) and natural killer (NK) cells (*n* = 8 and *n* = 7 patients) (Extended Data Fig. 3a). Disaggregated TILs were immediately frozen and subsequently thawed, stained and sorted at 4 °C directly into lysis buffer for RNA-seq, thereby maximizing preservation of the cells’ in situ transcriptomes.

**Extended Data Fig. 4:**
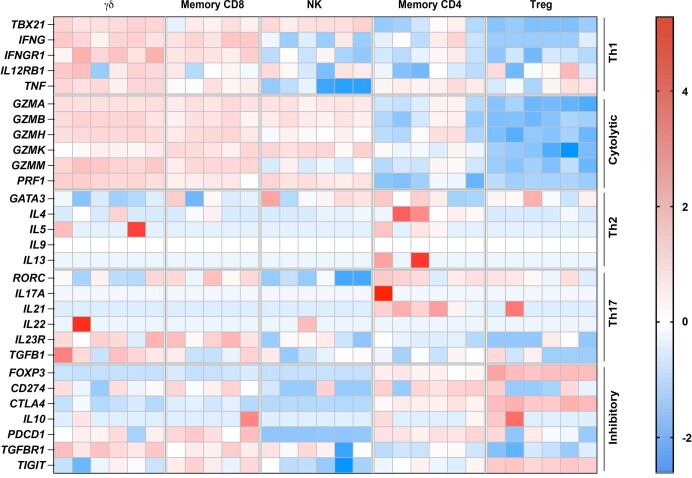
Peripheral blood γδ T cell functional transcriptomes. Expression of T cell master transcription factors and signature effector molecules of peripheral blood lymphocytes from the Blood Atlas project. Each column represents the denoted cell type from an individual donor. Colour scale denotes Z-score of Log2 (TPM + 1) of each gene. Source data

Cell types clustered together in a principal component analysis (PCA) of the 500 most variably expressed genes (Extended Data Fig. 3b) and expressed the anticipated canonical lineage markers (Extended Data Fig. 3b). Notably, Vδ1 T cells clustered together with CD8^+^ T cells and NK cells in this unsupervised analysis (Extended Data Fig. 3b). To determine the functional skew of Vδ1 T cells, we restricted our analysis to canonical transcription factors and effector molecules associated with T cell function and found that intratumoral Vδ1 T cells expressed the T_H_1 cell-specific master transcription factor Tbet (*TBX21*) as well as transcripts for granzymes (*GZM*s), perforin (*PRF1*) and IFN-γ (*IFNG*) to levels comparable with those of CD8^+^ T cells and NK cells (Fig. 3c). Moreover, intratumoral Vδ1 T cells did not express T_H_17 cell-associated genes or the T_H_17^−^ cell-specific master transcription factor RORγt (*RORC*). By contrast, these genes were expressed to a variable degree by co-located CD4^+^ T cells (Fig. 3c). To contextualize the in situ function of intratumoral Vδ1 T cells, we conducted a PCA using these restricted genes of interest expressed by intratumoral Vδ1, Vδ2, CD4^+^, CD8^+^ and T_reg_ cells and NK cells. The PCA demonstrated that intratumoral Vδ1 T cells transciptomically resemble CD8^+^ T cells and NK cells in function (Fig. 3d). To further contextualize our results, we reanalyzed expression of these genes in comparable cell types isolated from the peripheral blood of healthy volunteers as part of the Blood Atlas Project^39^ (Extended Data Fig. 4). Intratumoral Vδ1 T cells resemble peripheral blood γδ T cells in the expression of genes associated with T_H_1 cell, T_H_2 cell, cytolytic and inhibitory functions. Notably, peripheral blood γδ T cells showed some evidence for T_H_17 cell-associated gene expression (Extended Data Fig. 4), whereas this was not seen for intratumoral Vδ1 T cells (Fig. 3c). Most probably, T_H_17 cell-associated gene expression in blood reflected the predominance of Vδ2 T cells that have been reported to produce interleukin (IL)-17, albeit rarely^40–42^.

**Extended Data Fig. 5:**
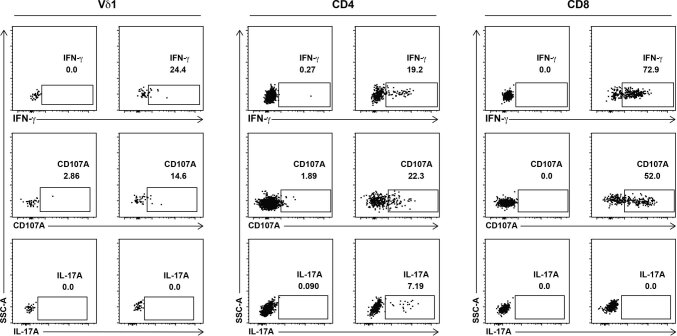
Effector function of *in vitro* stimulated TILs from NSCLCs. Representative flow cytometry dot plots of intracellular cytokine staining for IFN-γ and IL-17A and cell surface staining for CD107A in Vδ1, CD8 and CD4 T cells after *in vitro* stimulation of bulk tumour infiltrating lymphocytes with PMA and ionomycin (P-I). Gates were set on paired unstimulated negative controls (-). Percentage positive of parent population shown.

Next, we validated the functional potential of intratumoral Vδ1 T cells in vitro by stimulation of TILs with phorbol 12-myristate 13-acetate (PMA) and ionomycin, which mimics TCR signaling. Stimulated TILs were then stained for surface lineage markers and CD107A, a marker of cytotoxic degranulation, as well as for intracellular cytokines. Consistent with their gene expression profile, Vδ1 T cells produced IFN-γ and degranulated on activation (Fig. 3e and Extended Data Fig. 5). Moreover, we could find no evidence of IL-17A production by these cells in contrast to co-located CD4^+^ T cells (Fig. 3e and Extended Data Fig. 5).

**Extended Data Fig. 6:**
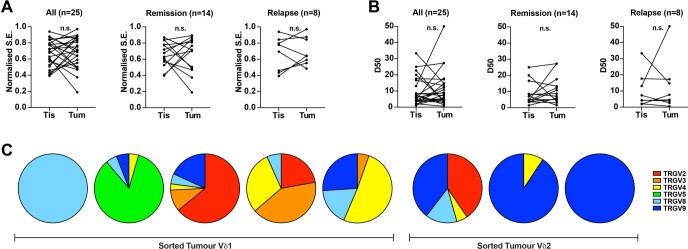
Tumour Vδ1 T cells demonstrate no evidence of clonotypic responses. **A)** Repertoire analysis by normalised Shannon entropy (S.E.) of Vδ1 T cells in NT tissue (Tis) and paired lung tumours (Tum) calculated on TCRδ CDR3 amino-acid sequences. Wilcoxon matched-pairs signed rank test. **B)** Repertoire analysis by D50 of Vδ1 T cells in NT tissue (Tis) and paired lung tumours (Tum) calculated on TCRδ CDR3 amino-acid sequences. Wilcoxon matched-pairs signed rank test. **C)** Relative expression (TPM) of functional TCRγ Vγ genes in sorted intra- tumoural Vδ1 T cells from 5 patients and Vδ2 from 3 patients. All datapoints represent independent patients. Source data

Previous studies have demonstrated that tissue-associated Vδ1 T cells may also be activated by the innate NKG2D receptor without requirement for contemporaneous TCR signaling^11,12,43^. Consistent with an innate, non-TCR/nonclonotypic response mode, we observed no significant clonal focusing of the TCRδ chain in Vδ1 T cells in tumors compared with NT tissues (Extended Data Fig. 6a,b). The gDNA−based TCRγ (TRG) sequencing from bulk tissues is inherently problematic because αβ T cells often harbor productive rearrangements of TRG genes^44^. Nevertheless, RNA-seq of sorted Vδ1 T cells from tumors, albeit in a limited cohort, demonstrated a diverse expression of Vγ chains in most patients, in contrast to Vδ2 T cells which predominantly employed Vγ9 (Extended Data Fig. 6c), further supporting a nonclonal innate response mode for Vδ1 T cells. Moreover, we also observed a positive and significant correlation of the presence of intratumoral Vδ1 T_EM_ cells with region-matched intratumoral expression of transcripts for NKG2D ligands (Fig. 3f).

### Intratumoral Vδ1 T cells demonstrate features of tissue residency and stemness

**Extended Data Fig. 7:**
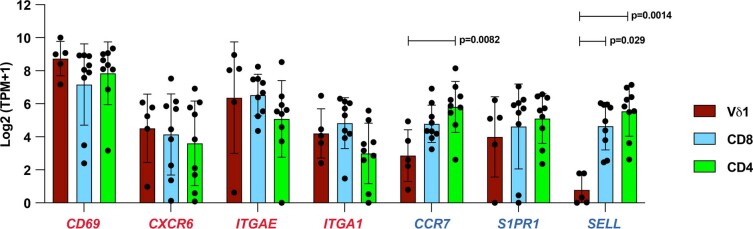
Tumour Vδ1 T cells possess a core T_RM_ signature. Expression of core TRM signature genes in Vδ1, CD8, and CD4 T cells sorted from tumours (n = 5, n = 9 and n = 9 patients respectively). Genes associated with tissue retention and homing and expected to be upregulated in TRM T cells highlighted in red. Genes associated with tissue egress and expected to be downregulated in TRM T cells highlighted in blue. Mean + /- S.D. plotted. Kruskal Wallis with post hoc Dunn’s test corrected for multiple testing. All datapoints represent independent patients. Source data

Whereas CD103 expression is often associated with T_RM_ status in T cells^45^, this is not always the case^46,47^. Seminal studies by independent teams have collectively established a core transcriptional profile of T_RM_ cells. Specifically, T_RM_ cells upregulate genes associated with tissue retention and homing (*CD69*, *CXCR6*, *ITGAE* and *ITGA1*) and downregulate genes associated with tissue egress (*CCR7*, *S1PR1* and *SELL*) compared with circulating T cells^48,49^. Thus, we compared the expression of these genes in intratumoral Vδ1 and CD8^+^ T cells, which were predominantly CD103^+^, versus intratumoral CD4^+^ T cells, which mostly lacked CD103 (Fig. 2b), and found that this tissue-resident profile was shared by the Vδ1 and CD8^+^ T cells, consistent with a bona fide T_RM_ status (Fig. 3g and Extended Data Fig. 7).

Recent studies have identified a distinct subset of stem-like memory CD8^+^ T cells in chronic infection and cancer defined by their expression of the transcription factors/regulators *EOMES*, *TCF7* and *TOX* and the IL-7 receptor (*IL7R*)^50–52^. These cells retain the capacity to proliferate despite chronic inflammatory stimuli and can give rise to highly functional effector cells implicated in tumor control and responses to programmed cell death protein 1 (PD-1) blockade^50,53^. Intratumoral Vδ1 T cells resembled stem-like CD8^+^ T cells in their expression of *EOMES*, *TCF7* and *TOX* distinct from intratumoral CD4^+^ T cells (Fig. 3h). Intratumoral Vδ1 T cells also expressed *IL7R* (Fig. 3h), albeit at a somewhat lower level, possibly because these cells are maintained by the epithelial-associated cytokine, IL-15 (ref. ^54^).

### Presence of Vδ1 T cells predicts ongoing remission in resected NSCLCs

**Fig. 4:**
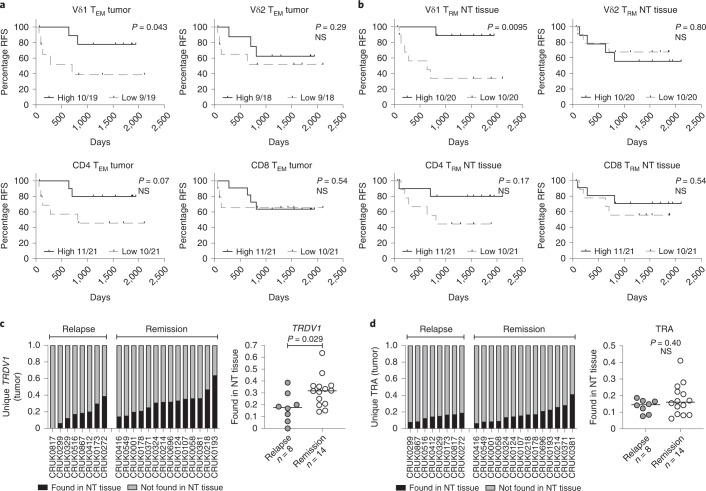
Presence of Vd1 T cells associates with RFS in resected NSCLCs. **a**, RFS split on median absolute numbers of Vδ1, Vδ2, CD4^+^ and CD8^+^ T_EM_ cells in tumors. The Gehan–Breslow–Wilcoxon test was used. **b**, RFS split on median absolute numbers of Vδ1, Vδ2, CD4^+^ and CD8^+^ T_RM_ cells in NT tissues. The Gehan–Breslow–Wilcoxon test was used. **c**, Proportion of unique Vδ1 (*TRDV1*) T cell clones present in tumors and also found in paired NT tissues. The bar represents the median. A two-tailed Mann–Whitney *U*-test was used. **d**, Proportion of unique αβ (TRA) T cell clones present in tumors and also found in paired NT tissues. The bar represents the median. A two-tailed Mann–Whitney *U*-test was used. Significant *P* values are shown. NS, not significant. The *n* numbers and datapoints represent independent patients. Source data

**Extended Data Fig. 8:**
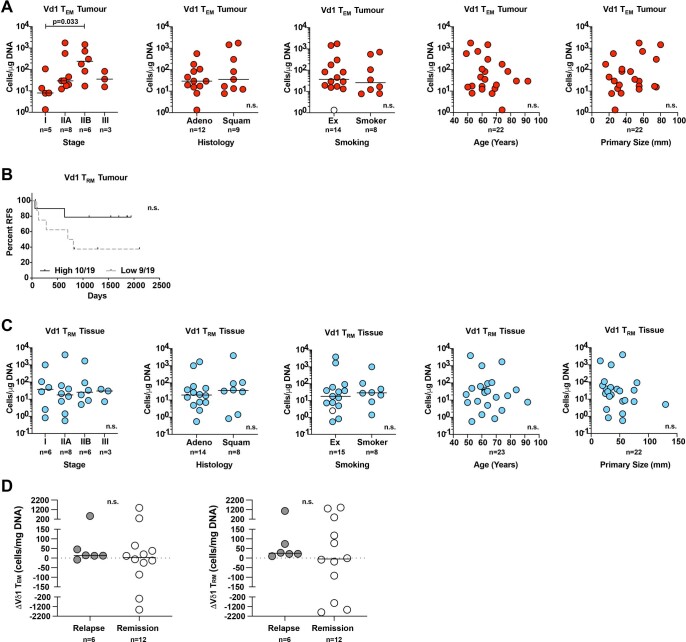
Prognostic value of Vδ1 T cells is largely independent of other prognostic clinicopathological features. **A)** Absolute numbers of Vδ1 TEM cells in tumours plotted by TNM stage, histology and smoking status (never smoker, unfilled circle) as well as against age and primary size. Kruskal Wallis with post hoc Dunn’s test corrected for multiple testing (stage). Bar=median. Mann-Whitney test (histology and smoking status). Spearman correlation (primary size and age). **B)** Relapse free survival split on median absolute numbers of Vδ1 Trm cells in tumours. Gehan-Breslow- Wilcoxon test. **C)** Absolute numbers of Vδ1 TEM cells in NT tissues plotted by TNM stage, histology and smoking status (never smoker, unfilled circle) as well as against age and primary size. Bar=median. Kruskal Wallis with post hoc Dunn’s test corrected for multiple testing (stage). Mann-Whitney test (histology and smoking status). Spearman correlation (primary size and age). **D)** ΔVδ1 TEM cells (absolute Vd1 TEM cells in tumour - absolute Vd1 TEM cells in paired NT tissue) and ΔVδ1 TRM cells (absolute Vd1 TRM cells in tumour - absolute Vd1 TRM cells in paired NT plotted by outcome. Bar=median. Mann-Whitney test. N numbers and datapoints represent independent patients. Source data

Given that Vδ1 T_RM_ and T_EM_ cells were enriched in tumors relative to NT lung tissues (see above), intratumoral Vδ1 T cells possess a Tc1 phenotype and these cells resemble stem-like CD8^+^ T cells, we examined their status in relation to clinical outcome in our cohort. Three patients who had incompletely excised primary tumors were excluded from this outcome analysis (Supplementary Table 1). To assess absolute numbers of T_RM_ (CD103^+^) and T_EM_ (CD45RA^−^CD27^−^) cells per unit of tissue/tumor, we mapped flow phenotyping proportions on to TCR-seq counts. Within tumors, the presence of Vδ1 T_EM_ cells was significantly associated with increased relapse-free survival (RFS) whereas this was only a trend for co-located Vδ2, CD4^+^ and CD8^+^ T_EM_ cells (Fig. 4a). Importantly, the association of intratumoral Vδ1 T_EM_ cells with improved RFS was not simply a reflection of less advanced disease because we found more intratumoral Vδ1 T_EM_ cells in advanced stages (Extended Data Fig. 8a). Furthermore, we found no association of intratumoral Vδ1 T_EM_ cells with primary tumor size, age, histology or smoking status (Extended Data Fig. 8a). The presence of Vδ1 T_RM_ cells in tumors trended toward association with increased RFS (Extended Data Fig. 8b). Conspicuously, we found the presence of Vδ1 T_RM_ cells in NT tissue to be highly and significantly associated with improved RFS, consistent with an epithelial immunosurveillance role proposed for these cells^16^ (Fig. 4b). There was no difference in the number of Vδ1 T_RM_ cells in NT tissue with regard to stage of disease, size of primary, age, histology or smoking status (Extended Data Fig. 8c).

Given the association of Vδ1 T cells in both NT tissues and tumors with ongoing remission, we asked whether such an association might exist for the delta (Δ) of the absolute numbers of Vδ1 T_EM_ cells and Vδ1 T_RM_ cells in NT tissues and paired tumors. No clear association of ΔVδ1 T_EM_ or ΔVδ1 T_RM_ cells with clinical outcome was observed (Extended Data Fig. 8d), suggesting that each subset, that is, intratumoral Vδ1 T_EM_ cells and NT tissue Vδ1 T_RM_ cells, has beneficial impacts independent of the status of their counterpart cells in the reciprocal tissue sites.

To explore further the contributions of tissue-resident Vδ1 T cells, we used the nucleotide sequence of the Vδ1 complementarity-determining region 3 (CDR3) as a molecular fingerprint to track unique Vδ1 T cell clones between NT tissues and paired tumors. On average, approximately a quarter of unique Vδ1 CDR3 sequences present in tumors were found in paired NT tissues (median, IQR: 27.3%, 16.5–35.4%) (Fig. 4c). By contrast, fewer unique TCRα clones present in tumors were also found in paired NT tissues (15.1%, 8.68–19.2%) (Fig. 4d), despite the lower potential for diversity in TCRα compared with TCRδ. Indeed, these data are consistent with a greater proportion of intratumoral αβ T cells (predominantly CD4^+^) being derived from peripheral blood as opposed to the tissue-resident pool that is the probable source of intratumoral Vδ1 T cells (Fig. 2b). When examined in relation to clinical outcome, patients with a greater proportion of intratumoral Vδ1 T cells also found in paired NT tissues were more likely to remain in remission, consistent with the cells’ proposed immunosurveillance function in steady-state tissues (Fig. 4c). Again, this was not the case for the αβ T cell compartment (Fig. 4d).

**Fig. 5:**
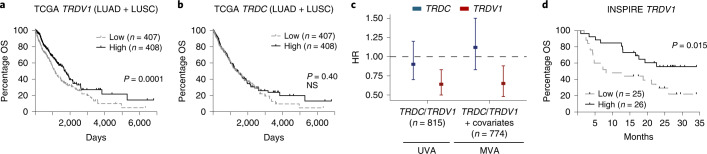
Expression of *TRDV1* gene predicts NSCLC survival in TCGA and survival post-pembrolizumab in advanced solid cancers. **a**, OS of patients with LUAD or LUSC in TCGA split on median *TRDV1* expression in primary tumor as a proxy for intratumoral Vδ1 T cells (*n* = 815 patients). The Gehan–Breslow–Wilcoxon test was used. **b**, OS of the same cohort of patients split on median *TRDC* expression as a proxy for intratumoral γδ T cells (*n* = 815 patients). The Gehan–Breslow–Wilcoxon test was used. NS, not significant. **c**, HRs for death in patients with above-median intratumoral expression of *TRDC* or *TRDV1* in UVA (*n* = 815 patients) and MVA (*n* = 774 patients) with age, gender, histology, smoking status, stage and *CD4* and *CD8B* gene expression. Rounded rectangles denote HRs and error bars denote 95% CIs. **d**, OS of patients with advanced solid cancers (mixed histologies) treated with pembrolizumab in the INSPIRE trial. There was a survival split on median *TRDV1* expression in primary tumor before pembrolizumab. The results were plotted in months after the third cycle of pembrolizumab. The Gehan–Breslow–Wilcoxon test was used. Significant *P* values are shown. Source data

Acknowledging the limited size of our cohort, we sought to validate our findings in a larger public dataset. The TCRδ locus is excised during TCRα rearrangement in αβ T cells^55^. Thus, we used the expression of *TRDV1* transcripts as a proxy for Vδ1 T cells and *TRDC* transcripts as a proxy for total γδ T cells, thereby assessing the association of these cells with NSCLC survival in The Cancer Genome Atlas (TCGA). Consistent with observations in our own cohort, when split on median expression, we found a significant association of high *TRDV1* expression with favorable overall survival (OS; Fig. 5a), although there was no association with *TRDC* expression (Fig. 5b). Moreover, the improved hazard ratio (HR) in patients with above-median *TRDV1* expression remained significant in multivariate analysis accounting for age, gender, histology, smoking status, stage and *CD4* and *CD8B* gene expression (HR = 0.65, 95% confidence interval (CI) = 0.48–0.88) (Fig. 5c).

**Extended Data Fig. 9:**
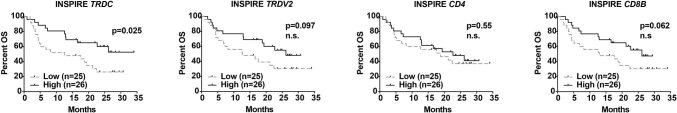
Association of survival with signature T cell lineage genes in the INSPIRE Trial. Overall survival of patients with advanced solid cancers (mixed histologies) treated with pembrolizumab in the INSPIRE trial. Survival split on median TRDC, TRDV2, CD4 and CD8B expression in primary tumour prior to pembrolizumab. Plotted in months after third cycle of pembrolizumab. Gehan-Breslow-Wilcoxon test. Significant p values shown. n.s. not significant. Source data

Finally, to explore whether there might be a role for Vδ1 T cells with respect to CPI therapy responses, we reanalyzed RNA-seq data from the INSPIRE trial (NCT02644369), a phase II basket clinical trial of pembrolizumab in advanced solid cancers^56^. Using *TRDV1* expression in pre-treatment tumor biopsies as a proxy for intratumoral Vδ1 T cells, we found that patients with above-median expression of *TRDV1* had significantly increased survival compared with those with below-median expression (Fig. 5d). Expression of *TRDC* (pan γδ T cells) was also predictive, although this was probably driven by Vδ1 T cells because the association of *TRDV2* expression (Vδ2T cells) with survival was only a trend (Extended Data Fig. 9). In addition, high expression of *CD4* (CD4^+^ T cells) did not associate with survival after pembrolizumab whereas high expression of *CD8B* (CD8^+^ T cells) trended toward association with improved survival (Extended Data Fig. 9).

## Discussion

Despite recent advances in immunotherapy, NSCLCs remain a leading cause of cancer-related mortality. Although these cancers are clearly susceptible to immunosurveillance, as evidenced by instances of durable responses to CPI therapy, only a minority of patients benefit. CPIs appear to work, at least in part, by de-repressing tumor (neo)antigen-specific αβ T cells. However, the body surfaces from which carcinomas arise are populated by many other immune cells. These compose organ-specific, tissue-resident immune compartments that collectively include considerably more T cells than the systemic lymphoid organs.

In mice, such compartments commonly include signature populations of tissue-resident γδ T cells seeded during fetal and perinatal development^20^. This is distinct from tissue-resident αβ T cells which arrive later in development, after priming in the lymph nodes as a response to infection^57^. Mice deficient in γδ T cells display heightened susceptibility to de novo cancers^16,58^, seemingly more so than mice deficient in αβ T cells^17^, establishing a critical and nonredundant role(s) for them. Indeed, these cells appear to be an essential early source of IFN-γ^59^, a cytokine pivotal for tumor rejection^26^. In addition to direct effector function, human γδ T cells are also capable of phagocytosis^60,61^ and professional antigen presentation to αβ T cells^60,62^. Such sentinel functions, traditionally associated with dendritic cells, are central to activating tumor (neo)antigen-specific αβ T cells and to orchestrating immune responses more generally^63^.

Despite apparently advantageous traits, γδ T cell immunotherapies have failed to demonstrate convincing efficacy in solid cancers including in NSCLCs^64^. However, those trials focused exclusively on the Vδ2 subset, the main subset of peripheral blood γδ T cells. By contrast, our assessments of tissue-resident lymphocytes in lung tissues have added to growing evidence that human body surfaces are primarily enriched for Vδ1 γδ T cells. Thus, these cells are well placed to detect malignancy vis-à-vis local/ipsilateral as well as metastatic/contralateral lung cancer recurrences, which comprise a considerable proportion of relapses after surgical resection.

Indeed, an increased presence of Vδ1 T_RM_ cells in NT lung tissues was predictive of ongoing remission in our cohort. Moreover, most Vδ1 T cells found within tumors were also of this tissue-resident phenotype, suggesting an improved capacity for tumor homing and/or retention. Strikingly, a greater proportion of shared Vδ1 T cell clones between tumors and NT tissues was significantly associated with remission in our cohort. Thus, this supports a potential patient-beneficial, cancer immunosurveillance role for human tissue-resident Vδ1 T cells that are present in steady-state NT lung tissues. Although many studies have demonstrated the prognostic utility of intratumoral TILs, these cells clearly cannot actively contribute to immunosurveillance after resection. Conversely, resident immune cells in juxtapositional normal tissues remain in situ at ‘ground-zero’ where they are well positioned to conduct ongoing cancer immunosurveillance.

Previous studies have demonstrated in vitro that Vδ1 T cells have a Tc1 phenotype^11,12,38,65^. However, given the scarcity of Vδ1 T cells, many of these studies have relied on ex vivo expansion using cytokine cocktails to achieve requisite numbers for in vitro assays. By using cells sorted directly from NSCLC tumors without ex vivo expansion or in vitro stimulation, we provide corroborating indirect and direct evidence for the in situ function of Vδ1 T cells through flow cytometric immunophenotyping and gene expression analysis. Compared with counterparts in NT lung tissues, Vδ1 T cells isolated from tumors were enriched in the CD45RA^−^CD27^−^ T_EM_ subset that has previously been described as potent IFN-γ producers^38^. Moreover, through gene expression analysis and in vitro functional assays, we demonstrate that intratumoral Vδ1 T cells are Tc1 skewed in situ.

Importantly, intratumoral Vδ1 T cells showed no evidence of a possibly tumor-promoting skew toward IL-17. Potentially dichotomous roles of γδ T cells in cancer immunosurveillance and immunotherapy are currently contentious^37^. Similar to αβ T cells, γδ T cells comprise distinct functional subsets. Thus, although absolute γδ T cell deficiency predisposes to cancer in mice, it is now increasingly clear that T_H_1-cell-skewed, IFN-γ-producing γδ T cells can be tumor rejecting^59^ whereas T_H_17-cell-skewed, IL-17-producing γδ T cells may be tumor promoting^18,66,67^. The limited evidence for IL-17 production by human γδ T cells, as opposed to murine γδ T cells, is confined mostly to a subset of peripheral blood-derived Vδ2 T cells^40–42^. By contrast, human Vδ1 T cells have been consistently demonstrated to display a tumor-rejecting Tc1 phenotype when activated in vitro^11,12,65^ and now we provide evidence of this in situ within the TME. Consistent with this, we found a significant association of intratumoral T_EM_ Vδ1 T cells with ongoing remission. Although this association was based on outcomes in a modest cohort of patients with early stage, surgically resected NSCLCs, we found a similar association between high *TRDV1* expression, as a proxy for intratumoral Vδ1 T cells, and survival in public TCGA data of ~800 patients with NSCLCs. Moreover, the association of Vδ1 T cells with favorable survival in TCGA was most evident in early-stage disease, alluding to the proposed role of γδ T cells as proximal immune sentinels of epithelial stress^59^. Our study does not directly address their role in metastatic disease where both the tumor-intrinsic biology and immune microenvironment, particularly at disparate metastatic sites, may be different. Likewise, we did not examine other immune compartments, such as the myeloid compartment or tertiary lymphoid structures, which may well contribute and warrant further study. Whether or not Vδ1 T cells directly effect tumor control or are merely a correlate of ongoing remission is difficult to answer without interventional studies such as adoptive Vδ1 T cell therapy, the first of which is currently under way for hematological cancers (NCT05001451). Nevertheless, the association of *TRDV1* expression with favorable responses to CPI therapy alludes to an important role for Vδ1 T cells. Indeed, a recent small study of mismatch repair-deficient colorectal cancers also implicated Vδ1 T cells in positive CPI responses^68^.

In considering the utilization of the Vδ1 subset of γδ T cells for cancer immunotherapy, one can now perceive several advantages over Vδ2 T cells. Vδ1 T cells are already resident in steady-state body surface tissues, raising the possibility that these cells may be beneficially manipulated in the clinic by off-the-shelf therapies, for example, via monoclonal antibodies targeting regulatory/stimulatory molecules^69^. Within tumors themselves, Vδ1 T cells display a tissue-resident CD103^+^ phenotype, potentially improving their tumor-homing and tumor-retention capabilities vis-à-vis adoptive cell therapy. Finally, Vδ1 T cells have been demonstrated by others to be less susceptible to activation-induced cell death, a key barrier to durable responses in adoptive cell therapy^70^.

## Methods

### Patients and samples

All clinical samples used were collected from patients recruited to the lung TRACERx Study (approved by an independent Research Ethics Committee, NRES Committee London, REC:13/LO/1546, https://clinicaltrials.gov/ct2/show/NCT01888601). All participants provided informed consent before taking part. Participants were not compensated. Tissue specimens were reviewed by a lung pathologist as previously described^22^. Of note, NT tissues were taken as far away as possible from tumors at primary surgery and H&E sections examined afterwards by a trained histopathologist to ensure samples that were tumor free. Samples were chosen based on the availability of banked TILs from NT lung tissues and paired tumors, region-matched bulk DNA and patients with at least one follow-up visit after surgical resection. Where available, banked contemporaneous PBMCs were also immunophenotyped by flow cytometry. No other selection criteria were applied. Fresh NT tissue and NSCLCs were finely minced with sterile scalpels and dissociated in type 1 collagenase (10 U ml^−1^, Thermo Fisher Scientific) and DNase I (75 μg ml^−1^, Roche) on a gentleMACS (Miltenyi Biotech) for 60 min at 37 °C. Digested material was passed through a 0.7-mm cell filter before TIL enrichment by Ficoll-paque gradient centrifugation (GE Healthcare). Isolated TILs were frozen in 10% dimethylsulfoxide (DMSO)/fetal calf serum (FCS) and stored in liquid nitrogen until analysis. PBMCs were isolated from whole blood by Ficoll-paque gradient centrifugation, frozen in FCS with 10% DMSO (v:v) and stored in liquid nitrogen until analysis. DNA from paired tumor regions and paired NT tissues for TCR-seq was extracted as previously described^24^. Briefly, region-matched tissues were homogenized using a TissueRuptor II (QIAGEN) and lysates passed through a QIAshredder column (QIAGEN) before DNA extraction using the Allprep DNA/RNA Mini kit (QIAGEN).

### Flow cytometry and FACS

Thawed samples were washed in sterile phosphate-buffered saline (PBS) to remove traces of DMSO and serum before staining with Zombie NIR viability dye (1:500 dilution in PBS for 15 min at room temperature). Samples were then stained for lineage and differentiation markers for 15 min at 4 °C (Supplementary Table 2; all antibodies used at 1:100 dilution in FACS buffer), washed twice with sterile 4 °C FACS buffer, kept on ice and immediately acquired on a BD LSRFortessa or sorted on a BD FACSAria Fusion, running BD FACSDiva, and exported as FCS3.0 files. FCS3.0 files were analyzed on FlowJo v.10. For RNA-seq (see below), cells were sorted directly into ~4 μl of lysis buffer (0.8% Triton X-100 in PBS (v:v) + 2 U ml^−1^ of RNase inhibitor) at 4 °C and lysates frozen at −80 °C. Analysis and results were based on populations with a parent gate of ten or more cells.

### In vitro activation assays

Thawed samples were washed in sterile PBS to remove traces of DMSO and rested overnight in complete RPMI medium (10% FCS + penicillin–streptomycin) at 37 °C and 5% CO_2_. Rested cells were seeded at up to 200,000 cells per well in 200 μl of complete RPMI medium the next day. Cells either received no stimulation (complete RPMI medium only) or were stimulated with PMA (10 ng ml^−1^) and ionomycin (1 μg ml^−1^). Brefeldin A (5 μg ml^−1^) and anti-CD107A (1:400 final dilution) were added to all the wells. Plates were centrifuged at 200*g* for 2 min and incubated at 37 °C and 5% CO_2_ for 5 h. After 5 h, cells were stained for surface lineage markers as described above. After surface staining, samples were fixed in BD CellFIX and washed twice with permeabilization wash buffer (BioLegend) before staining for intracellular cytokines (1:100 final dilution of each antibody in permeabilization wash buffer) for 20 min at 4 °C. Samples were then washed twice with permeabilization wash buffer, resuspended in FACS buffer and immediately acquired on a BD LSRFortessa.

### TCR-seq

The gDNA, 3 μg, extracted from region-matched tumors and NT tissues, was submitted for TCRα and TCRδ (TRA and TRD) sequencing with Adaptive Biotechnologies. Reads were aligned and annotated by Adaptive Biotechnologies. Sequences were filtered for in-frame CDR3 cells as well as TRA to TRA V-J family joins for αβ T cells and TRD to TRD V-J family joins for γδ T cells. Absolute counts of TCRs were normalized to 1 μg of input DNA for each sample to enable normalized comparison of T cell numbers across all samples. Data were analyzed using the immunoSEQ ANALYZER v.3.0 (Adaptive Biotechnologies).

### RNA-seq of sorted TIL populations, data processing and PCAs

Where sufficient material was available, NK cells and T cell subsets (Vδ1, Vδ2, CD4^+^, CD8^+^ and T_reg_ cells) were sorted (50–500 cells from each subset) from NT tissues and tumors from a cohort of donors (patient IDs: CRUK0230, CRUK0299, CRUK0329, CRUK0344, CRUK0412, CRUK0416, CRUK0516, CRUK0860, CRUK0844, CRUK0926, CRUK0949, CRUK0968, CRUK0914, CRUK0961 and CRUK0922). Given the limited availability of material, not all cell types could be sorted from the same samples. Sorted cell lysates were frozen at −80 °C and submitted to the Oxford Genomics Centre for low-input library preparation and sequencing. Library preparation was completed from sorted cells in lysate buffer using the Smart-Seq2 protocol with minor modifications and NexteraXT (Illumina) following the manufacturer’s instructions. Libraries were amplified (12 cycles) on a Tetrad (BioRad) using in-house dual indexing primers. Individual libraries were normalized using Qubit and the size profile was analyzed on the TapeStation 4200. Individual libraries were normalized and pooled together accordingly. The pooled library was diluted to ~10 nM for storage. The 10-nM library was denatured and further diluted before loading on the sequencer. Paired-end sequencing was performed using a HiSeq4000 75-bp platform (Illumina, HiSeq 3000/4000 PE Cluster Kit and 150-cycle SBS Kit), generating a raw read count of ~10 million reads per sample. The Trim Galore! utility v.0.4.2 (https://www.bioinformatics.babraham.ac.uk/projects/trim_galore—retrieved 3 May 2017) was used to remove sequencing adapters and quality trim individual reads with the *q*-parameter set to 20. Sequencing reads were aligned to the human genome and transcriptome (Ensembl GRCh38release-89) using RSEM v.1.3.0 (ref. ^71^) together with STAR aligner v.2.5.2 (ref. ^72^). Sequencing quality of individual samples was assessed using FASTQC v.0.11.5 (https://www.bioinformatics.babraham.ac.uk/projects/fastqc—retrieved 3 May 17) and RNA-SeQC v.1.1.8 (ref. ^73^). PCAs were generated using normalized counts of the top 500 variable genes filtered for an average transcripts per million (TPM) across all samples >1 (Extended Data Fig. 3b) or normalized counts of curated master transcription factors and effector molecules presented in Fig. 3c (Fig. 3d).

### Region-matched NKG2D ligand gene expression

For each region-matched sample, total RNA was extracted and prepared using a TruSeq Stranded Total RNA Human/Mouse/Rat ribo-depletion library preparation kit before Illumina complementary DNA paired-end sequencing. Libraries were prepared with ≥100 ng of total RNA input where possible and PCR amplified for 15 cycles. Libraries were quality checked by Agilent Tapestation and Promega QuantiFluor double-stranded DNA and pooled in equimolar amounts. Pooled libraries were sequenced in a HiSeq4000 at 50 million raw reads per sample, with a length of 75 bp or 100 bp per read. Illumina adapters were trimmed from raw reads using Cutadapt v.2.10 (ref. ^74^) with standard parameters. The quality of the trimmed reads was estimated per flow cell lane using FASTQC v.0.11.9. Fastq files with GC content within 2 s.d. of the cohort mean and <80% of total reads as duplicates were kept for alignment. These were then aligned to the UCSC hg19 human reference genome build using STAR aligner v.2.5.2a^72^ in two-pass mode with ENCODE 3 parameters generating one BAM file per tumor region. The same reads were also mapped to the human transcriptome (RefSeq GCA_000001405.1 build) using the same STAR parameters to generate gene expression data. Duplicates were marked with the MarkDuplicates function from GATK v.4.1.7.0 (ref. ^75^). Aligned reads were quality checked using QoRTs v.1.3.6 (ref. ^76^) for RNA integrity. Somalier v.0.2.7 (ref. ^77^) was used to detect potential instances of sample mislabeling. FASTQC, QoRTs and Somalier outputs were visualized using MultiQC v.1.9 (ref. ^78^). RSEM v.1.3.3 (ref. ^71^) was used with default parameters to quantify gene expression based on the BAM files aligned to the transcriptome. Gene expression patterns were used for further quality control of each sample. Tumor regions with <40% of all genes being expressed (>0 TPM) were excluded. In addition, samples with <20% of reads mapping uniquely to a single location in the genome were excluded.

### TCGA analysis

NSCLC data from TCGA were acquired using R software (v.4.0.2). Gene expression data, Workflow Type: HTSeq-Counts and clinical data from the TCGA-LUAD and TCGA-LUSC projects were downloaded from Genomic Data Commons (GDC) Data Portal using the R/Bioconductor package TCGAbiolinks v.2.16.4 (ref. ^79^). A total of 594 lung adenocarcinoma (LUAD) and 551 lung squamous cell carcinoma (LUSC) cases were retrieved. Clinical data were utilized to select for primary tumor samples and for patients with OS of at least 1 d, yielding 474 LUAD and 473 LUSC cases. Samples in which *TRDV1* transcript could be detected were included for analysis, thus yielding a final cohort of 417 LUAD and 398 LUSC cases (815 cases in total). Gene expression counts were normalized using DESeq2 v.1.28.1 (ref. ^80^) variance stabilizing transformation function and data were filtered for genes of interest: *TRDV1* (Vδ1 T cells) and *TRDC* (γδ cells). For survival analysis, patients were stratified into high- or low-expression groups for each gene of interest using median expression. OS was estimated from clinical data using ‘days_to_last_followup’ and ‘days_to_death’ and an OS event was defined from ‘vital_status’ (Dead/Alive).

The association of *TRDV1* and *TRDC* with survival outcomes in NSCLCs was further assessed by multivariate analysis (MVA) adjusted for age (continuous), gender (categorical), histology (categorical), smoking status (categorical), stage (ordinal), *CD4* expression (above/below median) and *CD8B* expression (above/below median) using the R package Survival (v.3.2-13). Of the variables assessed, only age, gender, histology, *CD4* expression and *CD8B* expression were available for all 815 cases. Smoking status was available for 782 cases and grouped into ‘Ex-smokers’, ‘Current Smokers’ and ‘Never smokers’ using ‘tobacco_smoking_history’. Patients with no smoking history information or current reformed smokers with no duration specified were excluded. Stage was available for 806 cases and grouped as stage 1, stage 2 and stage 3/4 owing to very few stage 4 cases. In total, 774 cases (out of 815 for *TRDV1/TRDC* univariate analysis (UVA)) were available for MVA.

### GTEx analysis

The data used for the analyses described in Extended Data Fig. 2 were obtained from the GTEx portal (www.gtexportal.org/) and GTEx Analysis Release v.8 (dbGaP accession no. phs000424.v8.p2) based on a search for *TRDC/TRDV1/TRDV2* without any further selection criteria. The data were accessed on 14 January 2022. Detailed methods for the GTEx project are available from the GTEx portal.

### Blood atlas analysis

The data used for analysis in Extended Data Fig. 5 were generated as part of the Blood Atlas Study^39^. Gene expression data were downloaded from https://www.proteinatlas.org/about/download on 14 January 2022. TPM values were extracted from ‘gdT cell’, ‘memory CD4 T cell’, ‘memory CD8 T cell’, ‘NK-cell’ and ‘T_reg_’ cell types, log_2_(TPM + 1) transformed and *z* normalized across each gene.

### INSPIRE survival analysis

Pre-processed RNA-seq and clinical data were downloaded for 51 patients in the INSPIRE cohort (NCT02644369) for whom both baseline gene expression and OS data were available. This pembrolizumab-treated cohort featured patients with a range of advanced solid cancers: head and neck (head and neck squamous cell cancers, *n* = 9), TNBCs (*n* = 6), high-grade serous carcinoma (*n* = 8), melanoma (*n* = 8) and other mixed solid tumors (*n* = 20). TPM values that had been log_2_(transformed) and batch normalized were downloaded as SourceData_Fig4.zip from Yang et al.^56^ and survival split on median expression of each gene.

### Statistics and reproducibility

No statistical method was used to predetermine sample size. Samples were chosen based on the availability of materials as described above. Three samples were excluded from outcome analysis due to involved margins on primary resection. The experiments were not randomized and investigators were not blinded to outcomes. The statistical tests used are indicated in the accompanying figure legends and two sided, where applicable, unless otherwise stated. Bonferroni’s correction was used to correct for multiple tests. All findings were considered significant at a *P*-value threshold of 0.05. Significant *P* values are indicated within the figures. Plots and graphs were generated with GraphPad Prism v.9 and JMP Pro v.15.

### Reporting Summary

Further information on research design is available in the Nature Research Reporting Summary linked to this article.

## Supplementary information

Supplementary Information Supplementary Table 1 Clinical features of patients. Supplementary Table 2 Key reagents and antibodies.

Reporting Summary

## Data Availability

The tumor region RNA-seq data, TCR-seq data and flow cytometry data (in each case from the TRACERx study) used or analyzed during the present study are available through the CRUK–University College London Cancer Trials Centre (ctc.tracerx@ucl.ac.uk) for academic noncommercial research purposes only, subject to review of a project proposal that will be evaluated by a TRACERx data access committee and any applicable ethical approvals, and entered into an appropriate data access agreement. Restrictions apply to the data availability to safeguard patient sequence data confidentiality, ensure compliance with patient study consent and meet data protection legislation, and due to commercial partnership requirements. Details of all public datasets obtained from third parties used in the present study are as follows. Blood Atlas Study (10.1126/science.aax9198) transcriptomic data were downloaded from https://www.proteinatlas.org/about/download. GTEx (www.gtexportal.org) Analysis Release v.8 was accessed via dbGaP (accession no. phs000424.v8.p2). INSPIRE trial (NCT02644369) transcriptomic data were downloaded as SourceData_Fig4.zip from Yang et al. (10.1038/s41467-021-25432-7). TCGA human LUAD and LUSC transcriptomic data were downloaded directly using the TCGAbiolinks R package derived from TCGA repository: https://portal.gdc.cancer.gov. Source data are provided with this paper.

## Source data

Source Data Fig. 1 Statistical source data.
Source Data Fig. 2 Statistical source data.
Source Data Fig. 3 Statistical source data.
Source Data Fig. 4 Statistical source data.
Source Data Fig. 5 Statistical source data.
Source Data Extended Data Fig. 1 Statistical source data.
Source Data Extended Data Fig. 3 Statistical source data.
Source Data Extended Data Fig. 4 Statistical source data.
Source Data Extended Data Fig. 6 Statistical source data.
Source Data Extended Data Fig. 7 Statistical source data.
Source Data Extended Data Fig. 8 Statistical source data.
Source Data Extended Data Fig. 9 Statistical source data.

## Extended data

Extended data are available for this paper at 10.1038/s43018-022-00376-z.

## Code availability

No customized code was used in the present study.

## References

[1] Allen EMV (2015). Genomic correlates of response to CTLA-4 blockade in metastatic melanoma. Science.

[2] Rizvi NA (2015). Mutational landscape determines sensitivity to PD-1 blockade in non-small cell lung cancer. Science.

[3] McGranahan N (2016). Clonal neoantigens elicit T cell immunoreactivity and sensitivity to immune checkpoint blockade. Science.

[4] Hellmann, M. D. et al. Nivolumab plus ipilimumab in lung cancer with a high tumor mutational burden. *N. Engl. J. Med.*10.1056/nejmoa1801946 (2018). 10.1056/NEJMoa1801946 PMC7193684 29658845

[5] Tumeh PC (2014). PD-1 blockade induces responses by inhibiting adaptive immune resistance. Nature.

[6] Sade-Feldman M (2018). Defining T cell states associated with response to checkpoint immunotherapy in melanoma. Cell.

[7] Shim, J. H. et al. HLA-corrected tumor mutation burden and homologous recombination deficiency for the prediction of response to PD-(L)1 blockade in advanced non-small-cell lung cancer patients. *Ann. Oncol*. **31**, 902–911 (2020). 10.1016/j.annonc.2020.04.004 32320754

[8] Ansell, S. M. et al. PD-1 blockade with nivolumab in relapsed or refractory Hodgkin’s lymphoma. *N. Engl. J. Med.*10.1056/nejmoa1411087 (2014). 10.1056/NEJMoa1411087 PMC4348009 25482239

[9] Hellmann MD (2018). Genomic features of response to combination immunotherapy in patients with advanced non-small-cell lung cancer. Cancer Cell.

[10] Gentles AJ (2015). The prognostic landscape of genes and infiltrating immune cells across human cancers. Nat. Med..

[11] Wu Y (2019). An innate-like Vδ1^+^ γδ T cell compartment in the human breast is associated with remission in triple-negative breast cancer. Sci. Transl. Med..

[12] Mikulak J (2019). NKp46-expressing human gut-resident intraepithelial Vδ1 T cell subpopulation exhibits high antitumor activity against colorectal cancer. JCI Insight.

[13] Foord E, Arruda LCM, Gaballa A, Klynning C, Uhlin M (2021). Characterization of ascites- and tumor-infiltrating γδ T cells reveals distinct repertoires and a beneficial role in ovarian cancer. Sci. Transl. Med..

[14] Zakeri N (2022). Characterisation and induction of tissue-resident gamma delta T cells to target hepatocellular carcinoma. Nat. Commun..

[15] Hayday AC, Vantourout P (2020). The innate biologies of adaptive antigen rReceptors. Annu. Rev. Immunol..

[16] Girardi M (2001). Regulation of cutaneous malignancy by gammadelta T cells. Science.

[17] Girardi M (2003). The distinct contributions of murine T cell receptor (TCR)γδ^+^ and TCRαβ^+^ T cells to different stages of chemically induced skin cancer. J. Exp. Med..

[18] Wu P (2014). γδT17 cells promote the accumulation and expansion of myeloid-derived suppressor cells in human colorectal cancer. Immunity.

[19] Daley D (2016). γδ T cells support pancreatic oncogenesis by restraining αβ T cell activation. Cell.

[20] Carding SR, Egan PJ (2002). Gammadelta T cells: functional plasticity and heterogeneity. Nat. Rev. Immunol..

[21] Craven KE, Gökmen-Polar Y, Badve SS (2021). CIBERSORT analysis of TCGA and METABRIC identifies subgroups with better outcomes in triple negative breast cancer. Sci. Rep..

[22] Jamal-Hanjani M (2017). Tracking the evolution of non-small-cell lung cancer. N. Engl. J. Med..

[23] Zappa C, Mousa SA (2016). Non-small cell lung cancer: current treatment and future advances. Transl. Lung Cancer Res..

[24] Rosenthal R (2019). Neoantigen-directed immune escape in lung cancer evolution. Nature.

[25] Alexandrov LB (2013). Signatures of mutational processes in human cancer. Nature.

[26] Shankaran V (2001). IFNγ and lymphocytes prevent primary tumour development and shape tumour immunogenicity. Nature.

[27] Gao J (2016). Loss of IFN-γ pathway genes in tumor cells as a mechanism of resistance to anti-CTLA-4 therapy. Cell.

[28] Rooney MS, Shukla SA, Wu CJ, Getz G, Hacohen N (2015). Molecular and genetic properties of tumors associated with local immune cytolytic activity. Cell.

[29] Pang DJ, Neves JF, Sumaria N, Pennington DJ (2012). Understanding the complexity of γδ T cell subsets in mouse and human. Immunology.

[30] Carithers LJ (2015). A novel approach to high-quality postmortem tssue procurement: the GTEx project. Biopreserv. Biobank.

[31] Farber DL, Yudanin NA, Restifo NP (2013). Human memory T cells: generation, compartmentalization and homeostasis. Nat. Rev. Immunol..

[32] Purwar R (2011). Resident memory T cells (T_RM_) are abundant in human lung: diversity, function, and antigen specificity. PLoS ONE.

[33] Snyder ME (2019). Generation and persistence of human tissue-resident memory T cells in lung transplantation. Sci. Immunol..

[34] Webb JR, Milne K, Nelson BH (2015). PD-1 and CD103 are widely coexpressed on prognostically favorable intraepithelial CD8 T cells in human ovarian cancer. Cancer Immunol..

[35] Savas P (2018). Single-cell profiling of breast cancer T cells reveals a tissue-resident memory subset associated with improved prognosis. Nat. Med..

[36] Djenidi F (2015). CD8^+^CD103^+^ tumor-infiltrating lymphocytes are tumor-specific tissue-resident memory T cells and a prognostic factor for survival in lung cancer patients. J. Immunol..

[37] Silva-Santos, B., Mensurado, S. & Coffelt, S. B. γδ T cells: pleiotropic immune effectors with therapeutic potential in cancer. *Nat. Rev. Cancer*10.1038/s41568-019-0153-5 (2019). 10.1038/s41568-019-0153-5 PMC7614706 31209264

[38] Davey MS (2017). Clonal selection in the human Vδ1 T cell repertoire indicates γδ TCR-dependent adaptive immune surveillance. Nat. Commun..

[39] Uhlen, M. et al. A genome-wide transcriptomic analysis of protein-coding genes in human blood cells. *Science* **366**, eaax9198 (2019). 10.1126/science.aax9198 31857451

[40] Ness-Schwickerath KJ, Jin C, Morita CT (2010). Cytokine requirements for the differentiation and expansion of IL-17A- and IL-22-producing human Vγ2Vδ2 T cells. J. Immunol..

[41] Tan L (2021). A fetal wave of human type 3 effector γδ cells with restricted TCR diversity persists into adulthood. Sci. Immunol..

[42] Caccamo N (2011). Differentiation, phenotype, and function of interleukin-17–producing human Vγ9Vδ2 T cells. Blood.

[43] Groh V, Steinle A, Bauer S, Spies T (1998). Recognition of stress-induced MHC molecules by intestinal epithelial γδ T cells. Science.

[44] Sherwood AM (2011). Deep sequencing of the human TCRγ and TCRβ repertoires suggests that TCRβ rearranges after αβ and γδ T cell commitment. Sci. Transl. Med..

[45] Szabo PA, Miron M, Farber DL (2019). Location, location, location: tissue resident memory T cells in mice and humans. Sci. Immunol..

[46] Bergsbaken T, Bevan MJ (2015). Proinflammatory microenvironments within the intestine regulate differentiation of tissue-resident CD8 T cells responding to infection. Nat. Immunol..

[47] Christo, S. N. et al. Discrete tissue microenvironments instruct diversity in resident memory T cell function and plasticity. *Nat. Immunol*. 10.1038/s41590-021-01004-1 (2021). 10.1038/s41590-021-01004-1 34426691

[48] Mackay LK (2013). The developmental pathway for CD103^+^CD8^+^ tissue-resident memory T cells of skin. Nat. Immunol..

[49] Kumar BV (2017). Human tissue-resident memory T cells are defined by core transcriptional and functional signatures in lymphoid and mucosal sites. Cell Rep..

[50] Im SJ (2016). Defining CD8^+^ T cells that provide the proliferative burst after PD-1 therapy. Nature.

[51] Alfei F (2019). TOX reinforces the phenotype and longevity of exhausted T cells in chronic viral infection. Nature.

[52] Eberhardt CS (2021). Functional HPV-specific PD-1^+^ stem-like CD8 T cells in head and neck cancer. Nature.

[53] Siddiqui I (2019). Intratumoral Tcf1^+^PD-1^+^CD8^+^ T cells with stem-like properties promote tumor control in response to vaccination and checkpoint blockade immunotherapy. Immunity.

[54] McCarthy NE, Eberl M (2018). Human γδ T cell control of mucosal immunity and inflammation. Front. Immunol..

[55] Villartay J-P, de Hockett RD, Coran D, Korsmeyer SJ, Cohen DI (1988). Deletion of the human T cell receptor δ-gene by a site-specific recombination. Nature.

[56] Yang SYC (2021). Pan-cancer analysis of longitudinal metastatic tumors reveals genomic alterations and immune landscape dynamics associated with pembrolizumab sensitivity. Nat. Commun..

[57] Schenkel JM, Masopust D (2014). Tissue-resident memory T cells. Immunity.

[58] Strid J (2008). Acute upregulation of an NKG2D ligand promotes rapid reorganization of a local immune compartment with pleiotropic effects on carcinogenesis. Nat. Immunol..

[59] Gao Y (2003). γδ T cells provide an early source of interferon γ in tumor immunity. J. Exp. Med..

[60] Wu Y (2009). Human gamma delta T cells: a lymphoid lineage cell capable of professional phagocytosis. J. Immunol..

[61] Junqueira, C. et al. γδ T cells suppress *Plasmodium falciparum* blood-stage infection by direct killing and phagocytosis. *Nat. Immunol*. 10.1038/s41590-020-00847-4 (2021). 10.1038/s41590-020-00847-4 PMC7906917 33432229

[62] Brandes M, Willimann K, Moser B (2005). Professional antigen-presentation function by human γδ T cells. Science.

[63] Hayday AC (2019). γδ T cell update: adaptate orchestrators of immune surveillance. J. Immunol..

[64] Kakimi K (2020). Adoptive transfer of zoledronate-expanded autologous Vγ9Vδ2 T cells in patients with treatment-refractory non-small-cell lung cancer: a multicenter, open-label, single-arm, phase 2 study. J. Immunother. Cancer.

[65] Almeida, A. R. et al. Delta one T cells for immunotherapy of chronic lymphocytic leukemia: clinical-grade expansion/ differentiation and preclinical proof-of-concept. *Clin. Cancer Res*. **22**, 5795–5804 (2016). 10.1158/1078-0432.CCR-16-0597 27307596

[66] Coffelt SB (2015). IL-17-producing γδ T cells and neutrophils conspire to promote breast cancer metastasis. Nature.

[67] Jin C (2019). Commensal microbiota promote lung cancer development via γδ T cells. Cell.

[68] Vries, N. L. de et al. γδ T cells are effectors of immune checkpoint blockade in mismatch repair-deficient colon cancers with antigen presentation defects. Preprint at *bioRxiv*10.1101/2021.10.14.464229 (2021).

[69] Payne KK (2020). BTN3A1 governs antitumor responses by coordinating αβ and γδ T cells. Science.

[70] Godder KT (2007). Long term disease-free survival in acute leukemia patients recovering with increased γδ T cells after partially mismatched related donor bone marrow transplantation. Bone Marrow Transpl..

[71] Li B, Dewey CN (2011). RSEM: accurate transcript quantification from RNA-Seq data with or without a reference genome. BMC Bioinf..

[72] Dobin A (2013). STAR: ultrafast universal RNA-seq aligner. Bioinformatics.

[73] DeLuca DS (2012). RNA-SeQC: RNA-seq metrics for quality control and process optimization. Bioinformatics.

[74] Martin M (2011). Cutadapt removes adapter sequences from high-throughput sequencing reads. EMBnet J.

[75] McKenna A (2010). The Genome Analysis Toolkit: a MapReduce framework for analyzing next-generation DNA sequencing data. Genome Res..

[76] Hartley SW, Mullikin JC (2015). QoRTs: a comprehensive toolset for quality control and data processing of RNA-Seq experiments. BMC Bioinf..

[77] Pedersen BS (2020). Somalier: rapid relatedness estimation for cancer and germline studies using efficient genome sketches. Genome Med..

[78] Ewels P, Magnusson M, Lundin S, Käller M (2016). MultiQC: summarize analysis results for multiple tools and samples in a single report. Bioinformatics.

[79] Colaprico A (2016). TCGAbiolinks: an R/Bioconductor package for integrative analysis of TCGA data. Nucleic Acids Res..

[80] Love MI, Huber W, Anders S (2014). Moderated estimation of fold change and dispersion for RNA-seq data with DESeq2. Genome Biol..

