# Supplementary material for: A local human Vδ1 T cell population is associated with survival in nonsmall-cell lung cancer
Source: Nat Cancer. 2022 May 30;3(6):696–709. doi: 10.1038/s43018-022-00376-z (PMC9236901; doi:10.1038/s43018-022-00376-z)
Supplement: Supplementary file 1 — Supplementary Table 1 Clinical features of patients. Supplementary Table 2 Key reagents and antibodies. [file 43018_2022_376_MOESM1_ESM.pdf]

nature portfolio

https://doi.org/10.1038/s43018-022-00376-z

Supplementary information

A local human Vδ1 T cell population is associated with survival in nonsmall-cell lung cancer

In the format provided by the
authors and unedited

Patient ID	Age	Sex	Smoking Status	Histology	Primary Size (mm)	Stage	Relapse	RFS (days)
CRUK0001	84	M	Ex-smoker	Adenocarcinoma	25	2A	N	1120
CRUK0058	74	M	Ex-smoker	Adenocarcinoma	16	2B	N	1872
CRUK0107	64	M	Ex-smoker	Squamous	80	2B	N	1862
CRUK0124^	78	M	Ex-smoker	Adenocarcinoma	35	2B	N	1544
CRUK0173	62	F	Ex-smoker	Squamous	40	3A	Y	812
CRUK0178	59	M	Ex-smoker	Adenocarcinoma	20	2A	N	66
CRUK0193	74	M	Never-smoker	Adenocarcinoma	26	1A	N	2107
CRUK0214	67	M	Smoker	Squamous	23	1B	N	833
CRUK0218	53	F	Smoker	Adenocarcinoma	34	1B	N	1291
CRUK0230#	53	F	Ex-smoker	Adenocarcinoma	55	2A	NA	NA
CRUK0271#	57	F	Smoker	Adenosquamous	59	2B	NA	NA
CRUK0272	92	M	Ex-smoker	Adenocarcinoma	55	2A	Y	95
CRUK0291#	62	M	Smoker	Squamous	50	3A	NA	NA
CRUK0299	49	F	Smoker	Adenocarcinoma	25	2A	Y	640
CRUK0324^	62	M	Smoker	Adenocarcinoma	29	1A	N	1886
CRUK0329	69	M	Ex-smoker	Squamous	30	1A	Y	703
CRUK0371^	87	M	Ex-smoker	Adenocarcinoma	18	1B	N	1429
CRUK0381^	52	F	Ex-smoker	Squamous	55	2A	N	1943
CRUK0412	50	M	Ex-smoker	Adenocarcinoma	75	3A	Y	280
CRUK0416	70	M	Ex-smoker	Adenocarcinoma	32	2A	N	99
CRUK0516*	60	M	Smoker	Squamous	73	2B	Y	59
CRUK0549^	64	F	Ex-smoker	Squamous	75	2B	N	1546
CRUK0696	60	F	Smoker	Adenocarcinoma	35	1B	N	1702
CRUK0817*	64	M	Ex-smoker	Adenocarcinoma	130	2B	Y	203
CRUK0867	60	M	Smoker	Squamous	44	2A	Y	131

Supplementary Table 1

Clinical features of patients. Cryopreserved PBMCs, NT tissue and tumour TILs were characterised by flow cytometry +/- TCRseq. ^Contemporaneous PBMC phenotyping not performed for these samples. #Involved margins on primary resection and thus excluded from outcome analysis. *Flow cytometry staining failed on TILs from CRUK516 NT tissue and CRUK817 tumour. “Ex-smoker” = stopped smoking for >12 months, “Smoker” = currently smoking or stopped smoking for <12 months. RFS: relapse-free survival.

Key Reagents		Supplier	Catalogue Number	Dilution Used
Anti-CD3	(PerCP-Cy5.5)	Biolegend	317336	1:100
Anti-CD4	(PE-Dazzle594)	Biolegend	317448	1:100
Anti-CD8a	(AF700)	Biolegend	300920	1:100
Anti-CD14	(PE-Cy7)	Biolegend	367112	1:100
Anti-CD19	(AF700)	Biolegend	363033	1:100
Anti-CD25	(BV650)	Biolegend	302634	1:100
Anti-CD27	(BV786)	BD Biosciences	563327	1:100
Anti-CD45	(BV605)	Biolegend	304042	1:100
Anti-CD45RA	(BV510)	Biolegend	304142	1:100
Anti-CD56	(APC)	Biolegend	362504	1:100
Anti-CD103	(BV421)	Biolegend	350214	1:100
Anti-CD107A	(BV650)	Biolegend	328637	1:100
Anti-IL-17	(APC)	Biolegend	512333	1:100
Anti-IFN-γ	(BV421)	Biolegend	502531	1:100
Anti-TCR Vδ1	(FITC)	ThermoFisher	TCR2730	1:100
Anti-TCR Vδ2	(PE)	Biolegend	331408	1:100
Anti-TCRγδ	(PE-Cy7)	Beckman Coulter	B10247	1:100
BD CellFIX		BD Biosciences	340181	1x
Brefeldin A		Biolegend	420601	5μg/ml
DNase		Roche	10104159001	75μg/mL
Permeabilisation Wash Buffer		Biolegend	421002	1x
Superase RNase Inhibitor		ThermoFisher	AM2694	2U/ml
Type 1 Collagenase		ThermoFisher	17018029	10U/ml
Zombie NIR Viability Dye		Biolegend	423106	1:500

Supplementary Table 2

Key reagents and antibodies.
