# Supplementary material for: A local human Vδ1 T cell population is associated with survival in nonsmall-cell lung cancer
Source: Nat Cancer. 2022 May 30;3(6):696–709. doi: 10.1038/s43018-022-00376-z (PMC9236901; doi:10.1038/s43018-022-00376-z)
Supplement: Supplementary file 2 — Reporting Summary [file 43018_2022_376_MOESM2_ESM.pdf]

nature portfolio

Corresponding author(s): Yin Wu
Adrian Hayday
Charles Swanton

Last updated by author(s): Mar 30, 2022

Reporting Summary

Nature Portfolio wishes to improve the reproducibility of the work that we publish. This form provides structure for consistency and transparency in reporting. For further information on Nature Portfolio policies, see our Editorial Policies and the Editorial Policy Checklist.

Statistics

For all statistical analyses, confirm that the following items are present in the figure legend, table legend, main text, or Methods section.

n/a	Confirmed	
☐	☒	The exact sample size (*n*) for each experimental group/condition, given as a discrete number and unit of measurement
☒	☐	A statement on whether measurements were taken from distinct samples or whether the same sample was measured repeatedly
☐	☒	The statistical test(s) used AND whether they are one- or two-sided<br>*Only common tests should be described solely by name; describe more complex techniques in the Methods section.*
☐	☒	A description of all covariates tested
☐	☒	A description of any assumptions or corrections, such as tests of normality and adjustment for multiple comparisons
☐	☒	A full description of the statistical parameters including central tendency (e.g. means) or other basic estimates (e.g. regression coefficient) AND variation (e.g. standard deviation) or associated estimates of uncertainty (e.g. confidence intervals)
☐	☒	For null hypothesis testing, the test statistic (e.g. *F*, *t*, *r*) with confidence intervals, effect sizes, degrees of freedom and *P* value noted<br>*Give P values as exact values whenever suitable.*
☒	☐	For Bayesian analysis, information on the choice of priors and Markov chain Monte Carlo settings
☒	☐	For hierarchical and complex designs, identification of the appropriate level for tests and full reporting of outcomes
☐	☒	Estimates of effect sizes (e.g. Cohen's *d*, Pearson's *r*), indicating how they were calculated

Our web collection on statistics for biologists contains articles on many of the points above.

Software and code

Policy information about availability of computer code

Data collection

No custom code was used to collect data.

NSCLC data from TCGA was acquired using R software (version 4.0.2). Gene expression data, Workflow Type: HTSeq-Counts and clinical data from the TCGA-LUAD and TCGA-LUSC projects were downloaded from Genomic Data Commons (GDC) Data Portal using the R/Bioconductor package TCGAbiolinks version 2.16.4.

BD FACSDiva software was used for collection of flow cytometry data

Data analysis

No custom code was used for data analysis.

CCutadapt version 2.10
DESeq2 version 1.28.185
FASTQC version 0.11.5
FASTQC version 0.11.9
GATK version 4.1.7.0
MultiQC version 1.9
QoRTs version 1.3.6
RNA-SeQC version 1.1.8
RSEM version 1.3.0
RSEM version 1.3.3
Somalier version 0.2.7
STAR aligner version 2.5.2
STAR aligner version 2.5.2a
Survival version 3.2-13

Trim Galore! utility version 0.4.2
FlowJo version 10
GraphPad Prism version 9.2.0
immunoSEQ Analyzer version 3.0
JMP Pro version 15.0.0

For manuscripts utilizing custom algorithms or software that are central to the research but not yet described in published literature, software must be made available to editors and reviewers. We strongly encourage code deposition in a community repository (e.g. GitHub). See the Nature Portfolio guidelines for submitting code & software for further information.

Data

Policy information about availability of data

All manuscripts must include a data availability statement. This statement should provide the following information, where applicable:

- Accession codes, unique identifiers, or web links for publicly available datasets
- A description of any restrictions on data availability
- For clinical datasets or third party data, please ensure that the statement adheres to our policy

The tumour region RNA-seq data, TCR-seq data, and flow cytometry data (in each case from the TRACERx study), used or analysed during this study are available through the Cancer Research UK & University College London Cancer Trials Centre (ctc.tracerx@ucl.ac.uk) for academic non-commercial research purposes only, subject to review of a project proposal that will be evaluated by a TRACERx data access committee and any applicable ethical approvals, and entering into an appropriate data access agreement. Restrictions apply to the data availability in order to safeguard patient sequence data confidentiality, to ensure compliance with patient study consent, to meet data protection legislation and due to commercial partnership requirements.

Details of all public datasets obtained from third parties used in this study are as follows. Blood Atlas Study (Uhlen et al. 2019, doi.org/10.1126/science.aax9198) transcriptomic data was downloaded from https://www.proteinatlas.org/about/download. GTEx (www.gtexportal.org/) Analysis Release V8 was accessed via dbGaP Accession phs000424.v8.p2. INSPIRE trial (NCT02644369) transcriptomic data were downloaded as SourceData_Fig4.zip from Yang et al. 2021, doi.org/10.1038/s41467-021-25432-7. TCGA human LUAD and LUSC transcriptomic data were downloaded directly using the TCGAbiolinks R package derived from TCGA repository https://portal.gdc.cancer.gov/.

Source data are available as Source Data files.

Field-specific reporting

Please select the one below that is the best fit for your research. If you are not sure, read the appropriate sections before making your selection.

☒ Life sciences ☐ Behavioural & social sciences ☐ Ecological, evolutionary & environmental sciences

For a reference copy of the document with all sections, see nature.com/documents/nr-reporting-summary-flat.pdf

Life sciences study design

All studies must disclose on these points even when the disclosure is negative.

Sample size	Samples (n=25) were chosen based on the availability of banked TILs from NT lung tissue and paired tumours, region-matched bulk DNA, and from patients with at least one follow-up visit after surgical resection. Where available, banked contemporaneous PBMCs were also immunophenotyped by flow cytometry. No other selection criteria were applied.
Data exclusions	Three patients were excluded from outcome analysis due to incomplete surgical resection of their primary tumours.
Replication	Survival outcome was validated in a cohort of 800+ patients with non-small cell lung cancer in the TCGA database. Technical replicates for flow cytometry, TCR sequencing, RNA sequencing and in vitro activation assays were not performed due to the limited availability of material. Biological replicates from multiple independent donors cross-validated the presence of gamma-delta T cell subsets and their phenotypes in clinical samples.
Randomization	Non-interventional study, not applicable
Blinding	Non-interventional study, not applicable

Reporting for specific materials, systems and methods

We require information from authors about some types of materials, experimental systems and methods used in many studies. Here, indicate whether each material, system or method listed is relevant to your study. If you are not sure if a list item applies to your research, read the appropriate section before selecting a response.

Materials & experimental systems

n/a	Involved in the study
☐	☒ Antibodies
☒	☐ Eukaryotic cell lines
☒	☐ Palaeontology and archaeology
☒	☐ Animals and other organisms
☐	☒ Human research participants
☐	☒ Clinical data
☒	☐ Dual use research of concern

Methods

n/a	Involved in the study
☒	☐ ChIP-seq
☐	☒ Flow cytometry
☒	☐ MRI-based neuroimaging

Antibodies

Antibodies used

Anti-CD3 (PerCP-Cy5.5) Biolegend Cat# 317336 Dilution 1:100
Anti-CD4 (PE-Dazzle594) Biolegend Cat# 317448 Dilution 1:100
Anti-CD8a (AF700) Biolegend Cat# 300920 Dilution 1:100
Anti-CD14 (PE-Cy7) Biolegend Cat# 367112 Dilution 1:100
Anti-CD19 (AF700) Biolegend Cat# 363033 Dilution 1:100
Anti-CD25 (BV650) Biolegend Cat# 302634 Dilution 1:100
Anti-CD27 (BV786) BD Biosciences Cat# 563327 Dilution 1:100
Anti-CD45 (BV605) Biolegend Cat# 304042 Dilution 1:100
Anti-CD45RA (BV510) Biolegend Cat# 304142 Dilution 1:100
Anti-CD56 (APC) Biolegend Cat# 362504 Dilution 1:100
Anti-CD103 (BV421) Biolegend Cat# 350214 Dilution 1:100
Anti-CD107A (BV650) Biolegend 328637 Dilution 1:400
Anti-IL-17 (APC) Biolegend 512333 Dilution 1:100
Anti-IFNγ (BV421) Biolegend 502531 Dilution 1:100
Anti-TCR Vδ1 (FITC) ThermoFisher Cat# TCR2730 Dilution 1:100
Anti-TCR Vδ2 (PE) Biolegend 3Cat# 31408 Dilution 1:100
Anti-TCRγδ (PE-Cy7) Beckman Coulter Cat# B10247 Dilution 1:100
Zombie NIR Viability Dye Biolegend Cat# 423106 Dilution 1:500

Validation

Antibodies and reagents used have been validated by commercial suppliers and validation statements can be found on suppliers' websites.

Biolegend
https://www.biolegend.com/en-us/quality/quality-control
"All of our products undergo industry-leading rigorous quality control (QC) testing to ensure the highest level of performance and reproducible results. Each lot is compared to an internally established "gold standard" to maintain lot-to-lot consistency. We also conduct wide-scale stability studies to guarantee an accurate shelf-life for our products. Additionally, we test the majority of our products on endogenous cells rather than transfected or immortal cells that may overexpress the analyte. We assess our reagents with samples and protocols that reflect our customers' experience. Our willingness to monitor the quality of our reagents extends beyond our lab and into yours."

BD Biosciences
https://www.bdbiosciences.com/en-gb/products/reagents/flow-cytometry-reagents/research-reagents/quality-and-reproducibility
"BD BIosciences not only develops its own antibodies but also collaborates with research scientists around the world to license their antibodies. We provide accessibility to the flow cytometry community by conjugating antibodies to a broad portfolio of high-performing dyes, including our vastly popular portfolio of BD Horizon BrilliantTM Dyes. A world-class team of research scientists helps ensure that these reagents work reliably and consistently for flow cytometry applications. The specificity is confirmed using multiple applications that may include a combination of flow cytometry, immunofluorescence, immunohistochemistry or western blot to test a combination of primary cells, cell lines or transfectant models. All flow cytometry reagents are titrated on the relevant positive or negative cells. To save time and cell samples for researchers, pre-titrated test size reagents are bottled at an optimal concentration, with the best signal-to-noise ratio on relevant models."

Beckman Coulter
https://www.mybeckman.uk/reagents/coulter-flow-cytometry
"Our portfolio of Flow Cytometry reagents, entirely manufactured under good manufacturing practices (GMP), covers major application areas including hemato-oncology, HIV analysis, immune monitoring, cell cycle and stem cells studies."

ThermoFisher
https://www.thermofisher.com/uk/en/home/life-science/antibodies/invitrogen-antibody-validation.html
"Part 1—Target specificity verification
This helps ensure the antibody will bind to the correct target. Our antibodies are being tested using at least one of the following methods to ensure proper functionality in researcher's experiments. Click on each testing method below for detailed testing strategies, workflow examples, and data figure legends.

Knockout—expression testing using CRISPR-Cas9 cell models
Knockdown—expression testing using RNAi to knockdown gene of interest
Independent antibody verification (IAV)—measurement of target expression is performed using two differentially raised antibodies

recognizing the same protein target
Cell treatment—detecting downstream events following cell treatment
Relative expression—using naturally occurring variable expression to confirm specificity
Neutralization—functional blocking of protein activity by antibody binding
Peptide array—using arrays to test reactivity against known protein modifications
SNAP-ChIP™—using SNAP-ChIP to test reactivity against known protein modifications
Immunoprecipitation-Mass Spectrometry (IP-MS)—testing using immunoprecipitation followed by mass spectrometry to identify antibody targets

Part 2—Functional application validation
These tests help ensure the antibody works in a particular application(s) of interest, which may include
(but are not limited to):

Western blotting
Flow cytometry
ChIP
Immunofluorescence imaging
Immunohistochemistry
Most antibodies were developed with specific applications in mind. Testing that an antibody generates acceptable results in a specific application is the second part of confirming antibody performance."

Human research participants

Policy information about studies involving human research participants

Population characteristics

This study includes a subset of patients recruited to the TRACERx Study for which population characteristics have previously been reported (DOI: 10.1056/NEJMoa1616288).

The population characteristics for the subset of patients included in this study are detailed in Supplementary Table 1

Recruitment

Samples (n=25) for this study were chosen based on the availability of banked TILs from NT lung tissue and paired tumours, region-matched bulk DNA, and from patients with at least one follow-up visit after surgical resection. Where available, banked contemporaneous PBMCs were also immunophenotyped by flow cytometry. No other selection criteria were applied. These criteria may potentially select for larger primary tumours within each stage and results should be interpreted with this in mind.

Ethics oversight

The study was approved by the NRES Committee London - Camden and Islington with the following details:
Study title: TRAcking non small cell lung Cancer Evolution through therapy (Rx)
REC reference: 13/LO/1546
Protocol number: UCL/12/0279
IRAS project ID: 138871

All participants provided informed consent prior to taking part.

Note that full information on the approval of the study protocol must also be provided in the manuscript.

Clinical data

Policy information about clinical studies
All manuscripts should comply with the ICMJE guidelines for publication of clinical research and a completed CONSORT checklist must be included with all submissions.

Clinical trial registration

TRACERx Lung: https://clinicaltrials.gov/ct2/show/NCT01888601, approved by an independent Research Ethics Committee, 13/LO/1546

INSPIRE Trial: https://clinicaltrials.gov/ct2/show/NCT02644369

Study protocol

TRACERx Lung: The study protocol is available as a supplement to Jamal-Hanjani et al. 2017, https://doi.org/10.1056/NEJMoa1616288

INSPIRE Trial: The study protocol is as a supplement to Yang et al. 2021, https://doi.org/10.1038/s41467-021-25432

Data collection

TRACERx Lung: Clinical and pathological data is collected from patients during study follow up - this period is a minimum of five years. Data collection is overseen by the sponsor of the study (Cancer Research UK & UCL Cancer Trials Centre) and takes place in hospitals across the United Kingdom. A centralised database called MACRO is used for this purpose. Data collection and recruitment commenced April 2014. Recruitment completed at all sites on December 16, 2022 except at London and Manchester hospital sites where recruitment is due to complete March 31, 2022. A full list of all sites for recruitment and data collection can be found in the study protocol (see above).

INSPIRE Trial: Patients recruitment and data collection was conducted at Princess Margaret Cancer Centre from March 21, 2016 to May 9, 2018. Data collection cutoff was July 18, 2019. Refer to Yang et al. 2021, https://doi.org/10.1038/s41467-021-25432

Outcomes

TRACERx Lung: disease-free survival is a pre-defined primary endpoint determined by RECIST v1.1 criteria. Last updated June 15, 2021.

INSPIRE Trial: Survival outcomes of the study was defined by progression free survival (PFS) and overall survival (OS). Clinical outcomes was determined by RECIST criteria and clinical benefit was defined as patients with complete or partial response or stable disease greater than 18 weeks. Refer to Yang et al. 2021, https://doi.org/10.1038/s41467-021-25432

Flow Cytometry

Plots

Confirm that:

☒ The axis labels state the marker and fluorochrome used (e.g. CD4-FITC).

☒ The axis scales are clearly visible. Include numbers along axes only for bottom left plot of group (a 'group' is an analysis of identical markers).

☒ All plots are contour plots with outliers or pseudocolor plots.

☒ A numerical value for number of cells or percentage (with statistics) is provided.

Methodology

Sample preparation	Thawed samples were washed in sterile PBS to remove traces of DMSO and serum before staining with Zombie NIR viability dye (1:500 dilution in PBS for 15min/room temperature). Samples were then stained for lineage and differentiation markers for 15 minutes at 4oC (see key reagents table, all antibodies used at 1:100 dilution in FACS buffer), washed twice with sterile 4oC FACS buffer, kept on ice and immediately acquired on a BD LSRFortessa or sorted on a BD FACSAria Fusion.<br><br>For in vitro activation assays, thawed samples were washed in sterile PBS to remove traces of DMSO and rested overnight in complete RPMI medium (10% FCS + penicillin/streptomycin) at 37oC/5% CO2. Rested cells were seeded at up to 200,000 cells/well in 200mcl of complete RPMI medium the following day. Cells received either no stimulation (complete RPMI medium only) or were stimulated with PMA (10ng/ml) and ionomycin (1mcg/ml). Brefeldin A (5mcg/ml) and anti-CD107A (1:400 final dilution) was added to all wells. Plates were centrifuged at 200g for 2 minutes and incubated at 37oC/5% CO2 for 5 hours. After 5 hours, cells were stained for surface lineage markers as described above. Following surface staining, samples were fixed in BD CellFIX and washed twice with permeabilization wash buffer (Biolegend) before staining for intracellular cytokines (1:100 final dilution of each antibody in permeabilization wash buffer) for 20 minutes at 4oC. Samples were then washed twice with permeabilization wash buffer, resuspended in FACS buffer and immediately acquired on a BD LSRFortessa.
Instrument	BD LSRFortessa or sorted on a BD FACSAria Fusion.
Software	FCS files were analysed using FlowJo version 10.
Cell population abundance	Between 50 and 500 cells were sorted for low input RNA sequencing as detailed in methods.
Gating strategy	Preliminary gates selected cells using an FSC and SSC gate. Doublets were excluded by plotting FSC area versus FSC height and excluding cells outside of a linear correlation. Live leukocytes were gated as CD45-high Zombie-NIR-low single cells. Subsequent gating was as described in supplementary data panels.

☒ Tick this box to confirm that a figure exemplifying the gating strategy is provided in the Supplementary Information.
